# Metabolic interplay between *Proteus mirabilis* and *Enterococcus faecalis* facilitates polymicrobial biofilm formation and invasive disease

**DOI:** 10.1128/mbio.02164-24

**Published:** 2024-10-30

**Authors:** Benjamin C. Hunt, Vitus Brix, Joseph Vath, Lauren Beryl Guterman, Steven M. Taddei, Namrata Deka, Brian S. Learman, Aimee L. Brauer, Shichen Shen, Jun Qu, Chelsie E. Armbruster

**Affiliations:** 1 Department of Microbiology and Immunology, Jacobs School of Medicine and Biomedical Sciences, State University of New York at Buffalo, Buffalo, New York, USA; 2 Department of Pharmaceutical Sciences, School of Pharmacy and Pharmaceutical Sciences, State University of New York at Buffalo, Buffalo, New York, USA; 3 NYS Center of Excellence in Bioinformatics and Life Sciences, Buffalo, New York, USA; Washington University School of Medicine, St. Louis, Missouri, USA; University of Notre Dame, Notre Dame, Indiana, USA

**Keywords:** *Proteus mirabilis*, *Enterococcus faecalis*, polymicrobial, biofilm, catheter-associated urinary tract infection, ornithine, arginine, bacterial metabolism, bacteremia, urinary tract infection

## Abstract

Biofilms play an important role in the development and pathogenesis of catheter-associated urinary tract infection (CAUTI). *Proteus mirabilis* and *Enterococcus faecalis* are common CAUTI pathogens that persistently co-colonize the catheterized urinary tract and form biofilms with increased biomass and antibiotic resistance. In this study, we uncover the metabolic interplay that drives biofilm enhancement and examine the contribution to CAUTI severity. Through compositional and proteomic biofilm analyses, we determined that the increase in biofilm biomass stems from an increase in the protein fraction of the polymicrobial biofilm. We further observed an enrichment in proteins associated with ornithine and arginine metabolism in polymicrobial biofilms compared with single-species biofilms. We show that arginine/ornithine antiport by *E. faecalis* promotes arginine biosynthesis and metabolism in *P. mirabilis*, ultimately driving the increase in polymicrobial biofilm protein content without affecting viability of either species. We further show that disrupting *E. faecalis* ornithine antiport alters the metabolic profile of polymicrobial biofilms and prevents enhancement, and this defect was complemented by supplementation with exogenous ornithine. In a murine model of CAUTI, ornithine antiport did not contribute to *E. faecalis* colonization but was required for the increased incidence of urinary stone formation and bacteremia that occurs during polymicrobial CAUTI with *P. mirabilis*. Thus, disrupting metabolic interplay between common co-colonizing species may represent a viable strategy for reducing risk of bacteremia.

**IMPORTANCE:**

Chronic infections often involve the formation of antibiotic-resistant biofilm communities that include multiple different microbes, which pose a challenge for effective treatment. In the catheterized urinary tract, potential pathogens persistently co-colonize for long periods of time and the interactions between them can lead to more severe disease outcomes. In this study, we identified the metabolite L-ornithine as a key mediator of disease-enhancing interactions between two common and challenging pathogens, *Enterococcus faecalis* and *Proteus mirabilis*. Disrupting ornithine-mediated interactions may therefore represent a strategy to prevent polymicrobial biofilm formation and decrease risk of severe disease.

## INTRODUCTION

Urinary tract infections (UTIs) are among the most common infections worldwide and account for approximately 40% of all nosocomial infections in the United States (1–5). Urinary catheterization is a common procedure in healthcare settings with approximately 15%–25% of hospital patients acquiring a catheter at some point in their stay; the incidence of catheterization is even more frequent for the elderly, long-term care patients, and critically ill patients (6–14). Catheter insertion facilitates the development of bacterial colonization through a variety of means, including providing an ideal surface for bacterial attachment (15–18). Each day a urinary catheter is in place, there is a compounding 3%–8% incidence of bacteriuria such that the majority of patients requiring long-term catheterization (>28 days) will experience continuous bacteriuria and at least one symptomatic catheter-associated UTI (CAUTI) (6, 12, 19–21).

CAUTIs involve a diverse range of pathogens, including *Escherichia coli*, *Proteus mirabilis*, *Enterococcus faecalis*, *Klebsiella* spp., *Pseudomonas aeruginosa*, and *Staphylococcus* species (5, 22–24). Catheter-associated bacteriuria and CAUTI are frequently polymicrobial, which further complicates treatment efficacy and infection severity (22, 25–28). With the rise in antimicrobial resistance and the growing appreciation for the polymicrobial nature of CAUTI, there is a clear need for investigations into the impact of polymicrobial interactions as they may result in synergistic effects for co-colonizing pathogens (29, 30).

Our prior work identified *P. mirabilis* and *E. faecalis* as the most common and persistent co-colonization partners in nursing home residents with long-term catheters (10, 22, 31), and this combination was also identified in 32% of catheterized individuals in an out-patient setting (28). *P. mirabilis* is Gram-negative, motile, and the most common cause of infection-induced urinary stones, catheter encrustation, and blockage (32–34). *E. faecalis* is Gram-positive, non-motile, highly antibiotic resistant, and of growing medical concern (35–37). We previously demonstrated that *P. mirabilis* and *E. faecalis* co-localize on catheters and within the bladder during experimental CAUTI, resulting in polymicrobial biofilms with enhanced biomass and antibiotic resistance (10). However, the underlying mechanism of biofilm enhancement was not elucidated. Coinfection of *P. mirabilis* with *E. faecalis* also dramatically increases the incidence of urolithiasis and bacteremia, although it is not yet known if increased disease severity is related to biofilm formation (38).

Two prior studies have found that *E. faecalis* constitutively secretes L-ornithine, thereby mediating metabolic cross-feeding that enhances growth and pathogenicity of *Clostridioides difficile* during gastrointestinal infection (39) and growth of *E. coli* under iron limitation during wound infection (40). In this study, we uncovered the contribution of ornithine-mediated metabolic interplay to polymicrobial biofilm enhancement and infection severity with *P. mirabilis* and *E. faecalis* in the context of the urinary tract. We demonstrate that secretion of L-ornithine from *E. faecalis* via the ArcD arginine/ornithine antiporter drives L-arginine biosynthesis and metabolism by *P. mirabilis*, ultimately increasing the protein content of polymicrobial biofilms and facilitating bacteremia. Thus, modulating the metabolic interplay between these species could potentially disrupt polymicrobial biofilm formation, persistent colonization, and risk of progression to severe disease.

## RESULTS

### *P. mirabilis* and *E. faecalis* polymicrobial biofilms have increased protein content

**Fig 1:**
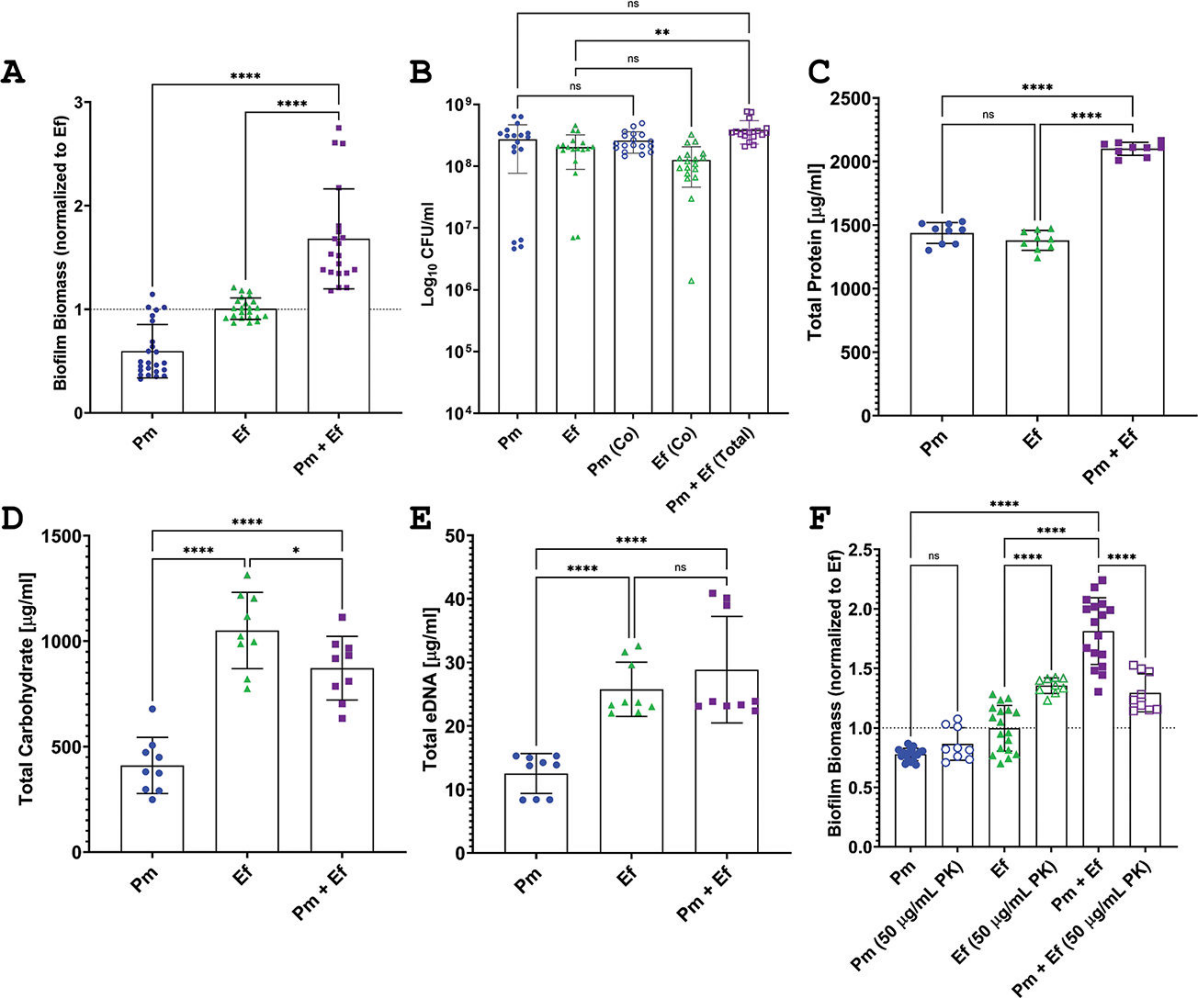
*P. mirabilis* and *E. faecalis* polymicrobial biofilms exhibit enhanced biofilm biomass characterized by an increase in protein content. (**A**) Crystal violet staining of biofilms grown for 24 hours in TSB-G. (**B**) CFUs of biofilms grown for 24 hours in TSB-G. (**C–E**) Compositional analysis of single or polymicrobial biofilms detailing total protein (**C**), total carbohydrate (**D**), and total extracellular DNA (eDNA) (**E**). For composition analysis, 24 replicate biofilms for each inoculum were suspended in a total volume of 3 mL water. (**F**) Crystal violet staining of biofilms grown with 50 µg/mL proteinase-K. Data represent the mean ± standard deviation (SD) for at least three independent experiments with at least two replicates each. ns, non-significant, *P < .05, **P < .01;, ***P < .001, and ****P < .0001 by one-way analysis of variance (ANOVA).

To investigate the underlying mechanism of enhanced biomass during polymicrobial biofilm formation, we began by studying single and polymicrobial biofilm formation in TSB-G (Tryptic soy broth supplemented with 1.5% glucose) under stationary conditions in 24-well plates. By crystal violet staining, single-species *E. faecalis* biofilms had slightly greater biomass than those formed by *P. mirabilis*, but co-culture significantly increased biofilm biomass compared with either species alone (Fig. 1A), confirming our previous observations (10). The increase in biofilm biomass was not driven by an increase in total bacterial burden as similar numbers of each species were recovered from single and co-culture biofilms (Fig. 1B).

We next quantified the amount of protein, carbohydrate, and extracellular DNA (eDNA) in the biofilms to identify the source of enhancement (Fig. 1C through E). Protein was the most abundant component overall and the only component of the polymicrobial biofilm that was significantly increased compared with each single-species biofilm (Fig. 1C through E), and the increase was derived specifically from the cell-associated fraction of the biofilm rather than the extracellular polymeric substance (Fig. S1A). While the carbohydrate and eDNA levels of the polymicrobial biofilm were higher than the *P. mirabilis* single-species biofilm, they were similar to the *E. faecalis* single-species biofilm and therefore not significantly enriched (Fig. 1D and E). The importance of protein in mediating the enhancement phenotype was confirmed by establishing biofilms in the presence of 50 µg/mL of proteinase K (PK), which had no effect on *P. mirabilis* biofilms, slightly increased *E. faecalis* biofilms, and significantly decreased biomass of polymicrobial biofilms (Fig. 1F).

### Polymicrobial biofilms are enriched in proteins pertaining to arginine/ornithine transport, biosynthesis, and metabolism

**Fig 2:**
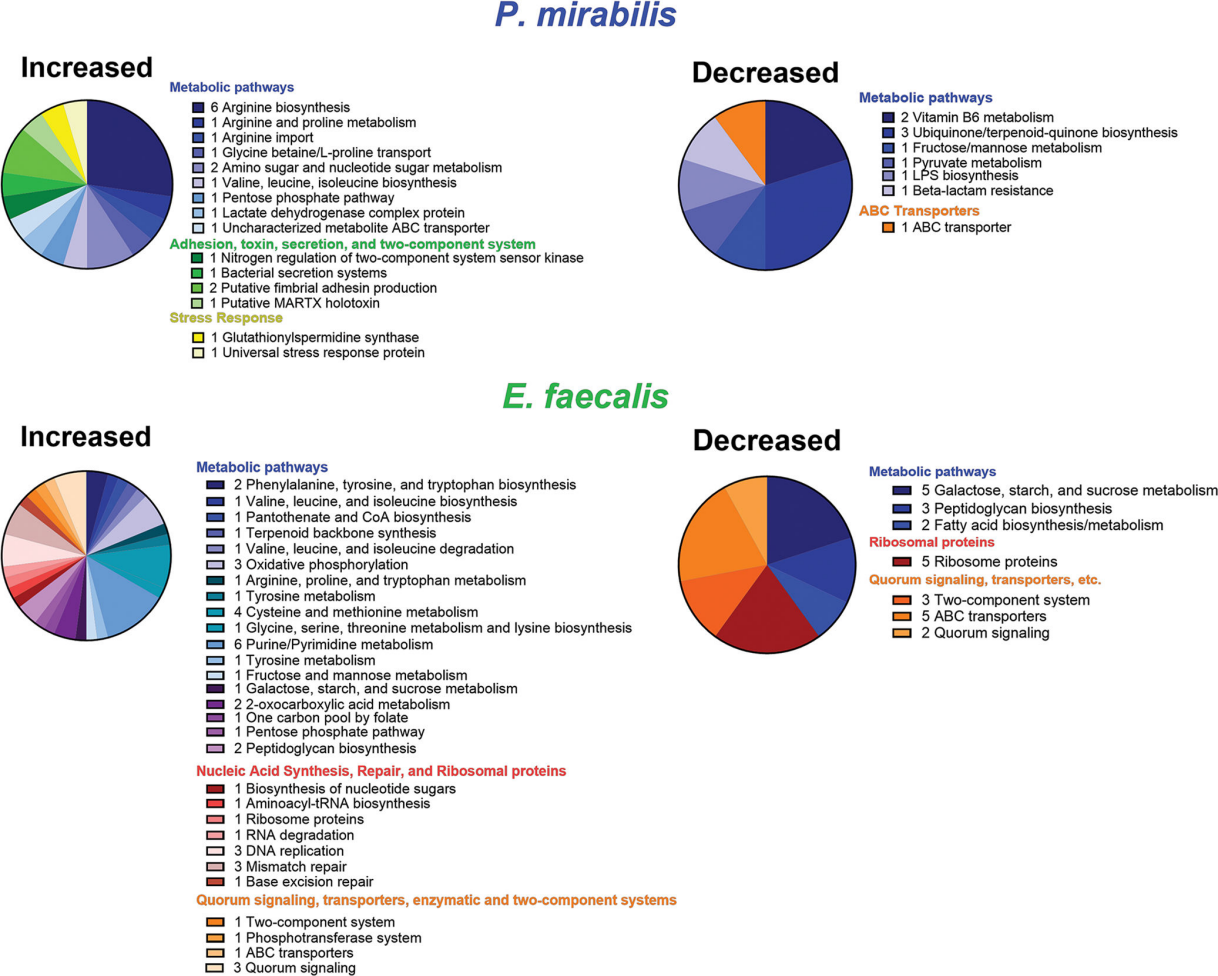
Proteins involved in amino acid import, biosynthesis, and metabolism exhibited differential abundance in polymicrobial biofilms compared with single biofilms. Pie charts display all proteins that mapped to KEGG pathways and were either increased or decreased >2-fold in polymicrobial biofilms compared with single-species biofilms. Proteins are grouped by broad functional categories. Note that some proteins mapped to multiple pathways and are therefore represented more than once in the pie charts. The top panels detail *P. mirabilis* proteins, while the bottom panels show *E. faecalis* proteins.

**Fig 3:**
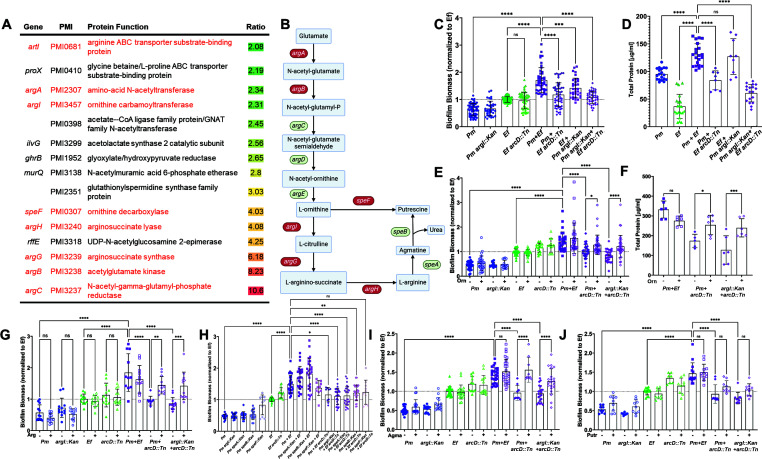
L-Ornithine secretion from *E. faecalis* drives *P. mirabilis* arginine biosynthesis and a contact-dependent increase in polymicrobial biofilm biomass. (**A**) Summary of *P. mirabilis* proteins involved in metabolism that were enriched within polymicrobial biofilms compared with single-species biofilms. Proteins related to ornithine metabolism, arginine biosynthesis, or transport are highlighted in red text. (**B**) All known genes involved in L-ornithine metabolism and L-arginine biosynthesis in *P. mirabilis* are displayed. L-Ornithine can either be directly catabolized to putrescine via ornithine decarboxylate (SpeF) or be fed into L-arginine biosynthesis via ornithine carbamoyltransferase (ArgI), which generates L-citrulline. Argininosuccinate synthase (ArgG) uses ATP to generate L-arginino-succinate from L-citrulline and L-aspartate; then, argininosuccinate lyase (ArgH) generates L-arginine and fumarate from L-arginino-succinate. L-Arginine can then be catabolized to putrescine via arginine decarboxylase (SpeA) and agmatinase (SpeB). (**C**) Crystal violet staining of single-species and polymicrobial biofilms grown for 24 hours in TSB-G. (**D**) Total protein content as measured by BCA from three pooled biofilms per experiment. (**E**) Crystal violet staining of biofilms grown for 24 hours in TSB-G with or without 10 mM of L-ornithine. (**F**) Total protein content as measured by BCA from three pooled biofilms per experiment when established in TSB-G with or without 10 mM of L-ornithine supplementation. (**G**) Crystal violet staining of biofilms grown for 24 hours in TSB-G with or without 10 mM of L-arginine. (**H**) Crystal violet staining of biofilms grown for 24 hours in TSB-G with *P. mirabilis* arginine catabolism mutants, *speA::Kan* and *speB::Kan*. (**I and J**) Crystal violet staining of biofilms grown for 24 hours in TSB-G with or without 10 mM of agmatine (**I**) or putrescine (**J**). Data represent the mean ± SD for three to five independent experiments with at least two replicates each. ns, non-significant, one-way ANOVA multiple comparisons, *P < 0.05, **P < 0.01, ***P < 0.001, and ****P < 0.0001.

By liquid chromatography mass spectrometry analysis, we identified an average of 1,296 proteins in *P. mirabilis* single-species biofilms, 869 in *E. faecalis* single-species biofilms, and 1,839 proteins in polymicrobial biofilms (675 *E. faecalis* and 1,164 *P. mirabilis*). Thus, total protein levels were confirmed to be higher in polymicrobial biofilms and 72%–85% was derived from *P. mirabilis* (Fig. S1B; Table S1). Analysis of proteins enriched in the polymicrobial biofilms (≥2-fold compared with single-species biofilm) revealed 240 *E. faecalis* proteins, of which 46 mapped to Kyoto Encyclopedia of Genes and Genomes (KEGG) pathways. Notably, 57% of the enriched proteins pertained to metabolic pathways and particularly biosynthesis and utilization of amino acids (Fig. 2; Table S1). Some proteins mapped to multiple KEGG pathways and are therefore represented more than once in the pie charts. Fewer *P. mirabilis* proteins were enriched overall in polymicrobial biofilms despite the observation that the majority of the polymicrobial protein content was derived from *P. mirabilis*. Of the 37 enriched *P. mirabilis* proteins, 15 mapped to KEGG pathways and 8 (53%) specifically pertained to arginine import, ornithine metabolism, and arginine biosynthesis (Fig. 2 and 3A; Table S1).

The enrichment of proteins pertaining to ornithine and arginine metabolism drew immediate interest in light of previous work by Keogh et al., wherein L-ornithine export from *E. faecalis* via the ArcD arginine/ornithine antiporter modulated biofilm formation, siderophore production, and fitness of *E. coli* (40). Another recent study demonstrated that ornithine and leucine produced by *E. faecalis* increased *Clostridioides difficile* fitness and pathogenesis in the gut (39). As displayed in Fig. 3B, *P*. *mirabilis* can either directly metabolize L-ornithine to the polyamine putrescine via ornithine decarboxylase (SpeF) or use L-ornithine for L-arginine biosynthesis via the combined actions of ornithine carbamoyltransferase (ArgI), argininosuccinate synthase (ArgG), and argininosuccinate lyase (ArgH). L-Arginine can then be used to produce putrescine via the sequential actions of arginine decarboxylase (SpeA) and agmatinase (SpeB).

Before investigating the importance of arginine/ornithine metabolism in mediating biofilm enhancement, we first examined the growth characteristics of the well-characterized *E. faecalis* OG1RF *arcD::Tn* transposon-insertion mutant as well as *P. mirabilis* ornithine catabolism mutants *speF::Kan* (PMI0307) and *argI::Kan* (PMI3457) (41–43). Disrupting ornithine export had no impact on *E. faecalis* growth or viability under any condition (Fig. S2A through C). Similarly, disrupting the ornithine metabolism pathway in *P. mirabilis* had no impact on growth or viability in rich media (Fig. S2D and E). However, in agreement with a recent publication (44), loss of *argI* resulted in arginine auxotrophy in minimal media (Fig. S2F). Auxotrophy was fully complemented by supplementation with either citrulline or arginine but not ornithine, agmatine, or putrescine, demonstrating that *argI* mediates the only route for L-arginine biosynthesis in *P. mirabilis* (Fig. S2F). Auxotrophy was also ablated by plasmid-based complementation with *argI* (Fig. S2G). In human urine that mainly contains amino acids and small peptides as nutrient sources (45) and low levels of arginine (~134 µM) (46), *P. mirabilis argI::Kan* plateaued after ~3 hours and was rescued by supplementation with L-citrulline (Fig. S2H). Thus, the inability to synthesize L-arginine imposes a ceiling on *P. mirabilis* growth in urine *in vitro*.

We also previously demonstrated that *P. mirabilis* and *E. faecalis* exhibit comparable growth rates during co-culture compared with single-species culture (38). However, considering the importance of L-arginine biosynthesis for *P. mirabilis* growth in minimal medium and urine, we sought to determine whether arginine/ornithine antiport in *E. faecalis* or arginine biosynthesis in *P. mirabilis* impact the viability of either species during co-culture in urine (Fig. S2I and J). Growth of *P. mirabilis argI::Kan* plateaued early during co-culture with wild-type *E. faecalis* (Fig. S2I), as was observed during single-species culture of *P. mirabilis argI::Kan* (Fig. S2H). However, growth of *P. mirabilis argI::Kan* did not plateau during co-culture with *E. faecalis arcD::Tn*, suggesting that disruption of arginine/ornithine antiport in *E. faecalis* may alter metabolite secretion in a way that counteracts arginine auxotrophy in *P. mirabilis*. Disrupting *arcD* also allowed *E. faecalis* to achieve a slightly faster initial growth rate during co-culture with *P. mirabilis* than wild-type *E. faecalis*, and this was independent of *P. mirabilis* L-arginine biosynthesis (Fig. S2J).

### L-Ornithine facilitates polymicrobial biofilm enhancement

To investigate the contribution of *E. faecalis* arginine/ornithine antiport to polymicrobial biofilm enhancement, we established single and polymicrobial biofilms with *P. mirabilis*, *P. mirabilis argI::Kan*, *E. faecalis*, and *E. faecalis arcD::Tn* and measured biofilm biomass and protein content. Neither of the mutants exhibited differences in single-species biofilm biomass compared with their respective parental strains (Fig. 3C). However, enhancement of biofilm biomass and protein content was abrogated during co-culture of *P. mirabilis* with *E. faecalis arcD::Tn* (Fig. 3C and D), indicating that arginine/ornithine antiport by *E. faecalis* is critical for the increased biomass that occurs during co-culture. When *P. mirabilis argI::Kan* was co-cultured with wild-type *E. faecalis*, biofilm enhancement was still observed but to a lower level than that for the parental strains and protein levels were similar. Thus, the ability of *P. mirabilis* to use L-ornithine for production of citrulline during L-arginine biosynthesis is not critical for biofilm enhancement under these conditions. Importantly, all differences in biofilm biomass and protein content were independent of any potential impact on bacterial viability (Fig. S3A).

We previously demonstrated that direct cell-cell contact was required for biofilm enhancement, as neither *P. mirabilis* nor *E. faecalis* exhibited altered biofilm biomass during co-culture when separated by a transwell insert (10). Thus, it was surprising that loss of arginine/ornithine antiport in *E. faecalis* abrogated polymicrobial biofilm enhancement. We therefore sought to determine if exogenous ornithine could promote biofilm enhancement. In agreement with our prior findings, the addition of 10 mM ornithine had no impact on single-species biofilm biomass for any of the strains (Fig. 3E). Ornithine supplementation also did not provide any further enhancement of the polymicrobial biofilm formed by the wild-type strains but increased biofilm biomass during co-culture of either wild-type *P. mirabilis* or *argI::Kan* with *E. faecalis arcD::Tn* to the level observed for co-culture of the wild-type strains (Fig. 3E). Importantly, ornithine supplementation also increased biofilm protein concentration to the level observed for co-culture of the wild-type strains (Fig. 3F). Thus, the presence of excess ornithine alone is sufficient to largely restore contact-dependent enhancement of biofilm biomass during co-culture. If ornithine-dependent arginine biosynthesis in *P. mirabilis* was required for biofilm enhancement, ornithine supplementation should not have restored enhancement during co-culture of *P. mirabilis argI::Kan* with *E. faecalis arcD::Tn*. Considering that auxotrophy of the *P. mirabilis argI::Kan* mutant could not be complemented by supplementation with ornithine, these findings suggest either that ornithine promotes biofilm enhancement through a mechanism that is independent of *P. mirabilis* arginine biosynthesis or that *P. mirabilis* has access to alternative precursors for arginine biosynthesis during co-culture with *E. faecalis*.

In addition to chemical complementation, we would ideally examine the impact of genetic complementation of *arcD* on biofilm enhancement during co-culture with *P. mirabilis*. However, polymicrobial biofilm studies posed a technical challenge for maintaining antibiotic-mediated plasmid selection. We therefore took a sequencing approach to identify any potential differences in the *E. faecalis arcD::Tn* mutant that could contribute to the co-culture phenotype outside of the function of *arcD*. The *arcD* gene is at the end of the *arcABCD* operon, which is transcribed in the opposite direction of the next downstream gene thereby making polar effects of transposon insertion unlikely, but other genomic alterations could still impact phenotype. As expected, a gene presence/absence analysis identified insertion of a chloramphenicol resistance gene and a transposon protein within the *arcD* gene as the only differences between the *arcD::Tn* mutant and our laboratory OG1RF wild-type strain (Table S2). We also examined single-nucleotide variations (SNVs) compared with the complete genome sequence of OG1RF in NCBI (BioSample SAMN02603002). Our wild-type OG1RF harbored three SNVs compared with the published genome: one nonsynonymous change in a PTS system transporter subunit, one additional A in a repetitive stretch in an acyl-ACP thioesterase domain-containing protein, and one additional T in a repetitive stretch in an intergenic region. The *arcD* transposon mutant also harbored the A and T repetitive region extra bases and one nonsynonymous SNV in an FAD-dependent oxidoreductase. Considering the very low number of SNVs and that supplementation with ornithine largely restored the defect of the *arcD* mutant during polymicrobial biofilm formation, it is likely that loss of *arcD* is the main factor responsible for the phenotype of the mutant.

To examine the specific contribution of arginine to biofilm enhancement, supplementation experiments were next repeated with 10 mM L-arginine (Fig. 3G). Supplementation again had no impact on single-species biofilms, but the addition of arginine restored biofilm enhancement during co-culture of either wild-type *P. mirabilis* or *argI::Kan* with *E. faecalis arcD::Tn*. Considering that *E. faecalis* encodes other arginine import systems such as the Art ABC transporter, excess arginine could still be taken up by *E. faecalis* without ornithine antiport. Thus, arginine import by either *P. mirabilis* or *E. faecalis* can also restore contact-dependent biofilm enhancement.

To determine if arginine catabolism or putrescine biosynthesis by *P. mirabilis* is required for biofilm enhancement, we next used *P. mirabilis* mutants in *speA*, *speB*, and *speF* (Fig. 3H) (47). Much like *P. mirabilis argI::Kan*, single-species biofilms formed by each of the mutants exhibited similar biomass to wild-type *P. mirabilis*. Polymicrobial biofilms formed with each of the mutants displayed biofilm biomass enhancement like wild-type *P. mirabilis*, although co-culture of *P. mirabilis speF::Kan* with *E. faecalis* resulted in less overall enhancement compared with the other biofilms. Collectively, these data indicate that L-arginine catabolism and putrescine biosynthesis are not required for polymicrobial biofilm enhancement. To confirm these results, we further examined the contribution of agmatine and putrescine to polymicrobial biofilm enhancement, as *E. faecalis* produces an agmatine/putrescine antiporter (48). Supplementation with 10 mM agmatine had no impact on single-species biofilms but fully restored contact-dependent biofilm enhancement during co-culture of either wild-type *P. mirabilis* or *argI::Kan* with *E. faecalis arcD::Tn* (Fig. 3I). In contrast, supplementation with putrescine failed to restore biofilm biomass (Fig. 3J). Since *P. mirabilis* can only use agmatine to produce putrescine and neither putrescine supplementation nor loss of agmatinase activity (*speB::Kan*) abrogated enhancement, our findings suggest that agmatine is most likely mediating enhancement via import by *E. faecalis*. Taken together, these data suggest that biofilm enhancement is mediated through a combination of ornithine production by *E. faecalis*, agmatine import by *E. faecalis*, and arginine import by at least one species, all of which are disrupted by loss of arginine/ornithine antiport in *E. faecalis*.

### Metabolic analysis of biofilm supernatants reveals temporal changes in arginine and ornithine levels

**Fig 4:**
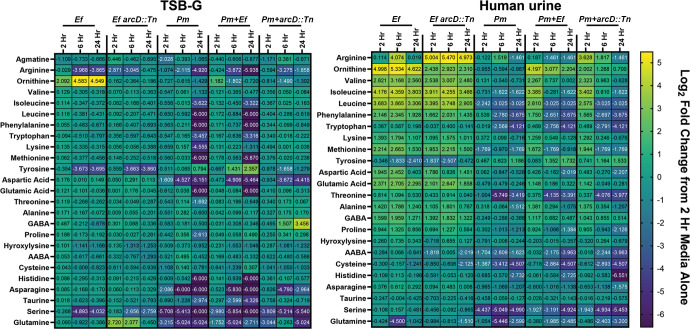
Metabolite profiles of single and polymicrobial biofilms established in TSB-G or pooled human urine. Metabolomics analysis of supernatants collected from single and polymicrobial biofilms at 2, 6, and 24 hours post-inoculation in either TSB-G or dilute human urine. Average metabolite concentrations from two independent experiments are represented as log_2_ fold change compared with an uninoculated media-alone control (either TSB-G or urine) collected at the 2-hour time point.

Our results thus far indicate that arginine/ornithine antiport by *E. faecalis* is critical for contact-dependent polymicrobial biofilm enhancement during co-culture with *P. mirabilis* in TSB-G. Importantly, when biofilms were established in human urine instead of TSB-G, complete abrogation of biofilm enhancement required disruption of both *P. mirabilis argI::Kan* and *E. faecalis arcD::Tn* (Fig. S3B), suggesting media-specific differences in metabolism. We therefore took a metabolomics approach to further examine metabolic interplay between *P. mirabilis* and *E. faecalis* during biofilm formation in TSB-G and human urine (Fig. 4). Biofilm supernatants were collected at 2, 6, and 24 hours for quantification of amino acids by UPLC, and data were expressed as log_2_ fold change over sterile media incubated under the same conditions.

In TSB-G, the only major differences in the metabolite profile of the *E. faecalis arcD::Tn* mutant compared with the parental strain pertained to ornithine and arginine levels; ornithine levels increased over time in supernatants from wild-type biofilms while arginine levels decreased (Fig. 4; Table S3). In contrast, ornithine levels remained unchanged in supernatants from *E. faecalis arcD::Tn* while arginine levels were initially depleted and then replenished. Glutamine levels were also significantly higher in supernatants from *E. faecalis arcD::Tn* at 2 and 6 hours. Supernatants from *P. mirabilis* single-species biofilms exhibited a very different metabolite profile than *E. faecalis*, with the majority of amino acids being depleted by 24 hours (Fig. 4; Table S3). One notable exception was tyrosine, which was initially decreased at 2 hours but increased by 24 hours. Supernatants from co-culture of *P. mirabilis* with wild-type *E. faecalis* largely mirrored the profile of *P. mirabilis* alone, although ornithine levels were increased at 2 hours before being depleted, arginine levels depleted faster, and tyrosine levels were further increased by 24 hours. When polymicrobial biofilms were instead formed with *E. faecalis arcD::Tn*, ornithine levels were consistently low, arginine was not depleted as extensively, and tyrosine did not accumulate. Furthermore, disrupting *arcD* resulted in significantly higher levels of several amino acids at 24 hours (leucine, isoleucine, phenylalanine, tryptophan, methionine, glutamic acid, histidine, asparagine, taurine, and GABA; Table S3). Thus, disrupting arginine/ornithine antiport by *E. faecalis* has a substantial impact on amino acid import, biosynthesis, and metabolism in polymicrobial biofilms.

Interestingly, disrupting either arginine biosynthesis (*argI*) or arginine catabolism (*speA* and *speB*) had minimal impact on the metabolite profile of *P. mirabilis* single-species biofilms (Table S3, Fig. S4).). Loss of *argI* resulted in less-pronounced depletion of phenylalanine, lysine, and histidine at 24 hours, and this was largely recapitulated in polymicrobial biofilms formed by *P. mirabilis argI::Kan* with wild-type *E. faecalis*. Loss of *speA* similarly resulted in less-pronounced depletion of lysine, leucine, methionine, and cysteine at 24 hours for single-species biofilms but also disrupted depletion of isoleucine, phenylalanine, tryptophan, glutamic acid, histidine, and taurine in polymicrobial biofilms formed with wild-type *E. faecalis*. Thus, arginine catabolism by *P. mirabilis* appears to contribute to metabolic interplay with wild-type *E. faecalis*.

Overall, ornithine was specifically observed to accumulate in supernatants from wild-type *E. faecalis* single-species biofilms and it was depleted in all other conditions. Loss of *arcD* decreased arginine depletion under all conditions, including all co-cultures. With respect to polymicrobial biofilms, loss of *arcD* prevented tyrosine accumulation, decreased glutamic acid depletion, and facilitated GABA accumulation, and this was independent of arginine biosynthesis or metabolism by *P. mirabilis*. However, disrupting either arginine/ornithine antiport in *E. faecalis* or arginine biosynthesis/metabolism in *P. mirabilis* abrogated methionine and histidine depletion.

When biofilms were instead established in pooled human urine (Fig. 4), both *E. faecalis* strains produced numerous amino acids including valine, isoleucine, leucine, phenylalanine, and glutamic acid. The main difference between *E. faecalis arcD::Tn* and the wild type again pertained to ornithine and arginine levels, as expected (Table S3). Similar to the TSB-G metabolite profiles, supernatants from *P. mirabilis* single-species biofilms generally exhibited amino acid depletion but a slight increase in tyrosine levels. Co-culture of *P. mirabilis* with wild-type *E. faecalis* again largely mirrored the profile of the *P. mirabilis* biofilm, except amino acid depletion was not as robust at 2 hours and ornithine levels remained consistently high. When polymicrobial biofilms were formed with *E. faecalis arcD::Tn*, the only differences were a reduction in ornithine levels and an initial increase in arginine levels that was significant at 2 hours only (Table S3). Thus, ornithine and arginine are the only amino acids for which supernatant concentrations consistently differed during polymicrobial biofilm formation, underscoring the importance of arginine/ornithine interplay to *P. mirabilis* and *E. faecalis* interactions. It is also notable that tyrosine and GABA levels did not substantially differ between polymicrobial biofilms formed in urine, suggesting a specific influence of media composition.

### Metabolic interplay between *E. faecalis* and *P. mirabilis* contributes to catheter biofilm formation

**Fig 5:**
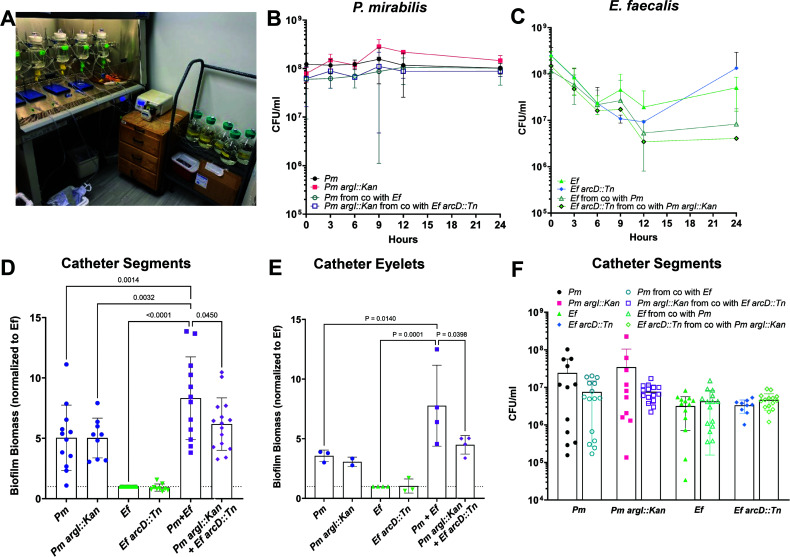
Arginine/ornithine antiport by *E. faecalis* and L-arginine biosynthesis by *P. mirabilis* contribute to enhanced biofilm biomass on silicone catheters under flow conditions. (**A**) Image of the glass bladder model setup with five bladders running in tandem. (**B and C**) CFUs of *P. mirabilis* (**B**) or *E. faecalis* (**C**) wild-type and mutant bacteria were enumerated from effluent collected through the catheter port for each bladder at 0, 3, 6, 9, 12, and 24 hours post-inoculation. (**D and E**) Crystal violet staining of bacterial biomass on either 10-mm catheter segments (**D**) or the catheter eyelet (**E**) 24 hours post-inoculation. (**F**) CFUs enumerated from 10-mm catheter segments at 24 hours post-inoculation. Data represent mean ± SD for at least three independent glass bladder experiments with three replicate catheter segments each. ns, non-significant, one-way ANOVA multiple comparisons, *P < 0.05, **P < 0.01, ***P < 0.001, and ****P < 0.0001.

To further examine the contribution of *E. faecalis* arginine/ornithine antiport and *P. mirabilis* arginine biosynthesis to biofilm formation on physiologically relevant surfaces under flow conditions, we utilized a glass bladder model of CAUTI. A picture of five bladders running in tandem is shown in Fig. 5A. Since each bladder requires 2 L of medium every 24 hours, these experiments were conducted using artificial urine media (AUM). No differences in viability of any strain was observed in the AUM effluent collected from the catheter port aside when grown individually, although there was a ~ 1 log decrease in *E. faecalis* CFUs during polymicrobial infection that could be due to changes in pH driven by *P. mirabilis* urease activity in AUM (Fig. 5B and C). At 24 hours post-inoculation, biofilm biomass and CFUs were examined across the length of catheter as well as biomass on the catheter eyelet. Co-infection with the parental strains resulted in significant biomass enhancement on catheter segments (Fig. 5D) and eyelets (Fig. 5E) compared with single-species biofilms, and enhancement was absent during coinfection of *P. mirabilis argI::Kan* with *E. faecalis arcD::Tn*. Changes in biofilm biomass were not driven by changes in viability as there were no significant differences between CFUs (Fig. 4F). Thus, arginine/ornithine metabolic interplay contributes to polymicrobial biofilm enhancement under physiological conditions.

  We previously demonstrated that co-culture with *E. faecalis* increases *P. mirabilis* urease activity, which could contribute to increased catheter biofilm biomass due to ion precipitation and catheter encrustation (38). We therefore examined overall urease activity in *P. mirabilis argI::Kan* as well as *E. faecalis*-mediated enhancement (Fig. S5). Urease activity was unaltered in *P. mirabilis argI::Kan* compared with wild-type *P. mirabilis*, and supernatants taken from either wild-type *E. faecalis* or *arcD::Tn* enhanced activity in both *P. mirabilis* strains to a similar degree. Thus, arginine/ornithine-mediated metabolic interplay between *P. mirabilis* and *E. faecalis* facilitates catheter biofilm enhancement independent of any potential impact on urease activity.

### Metabolic interplay contributes to bacteremia during polymicrobial infection

**Fig 6:**
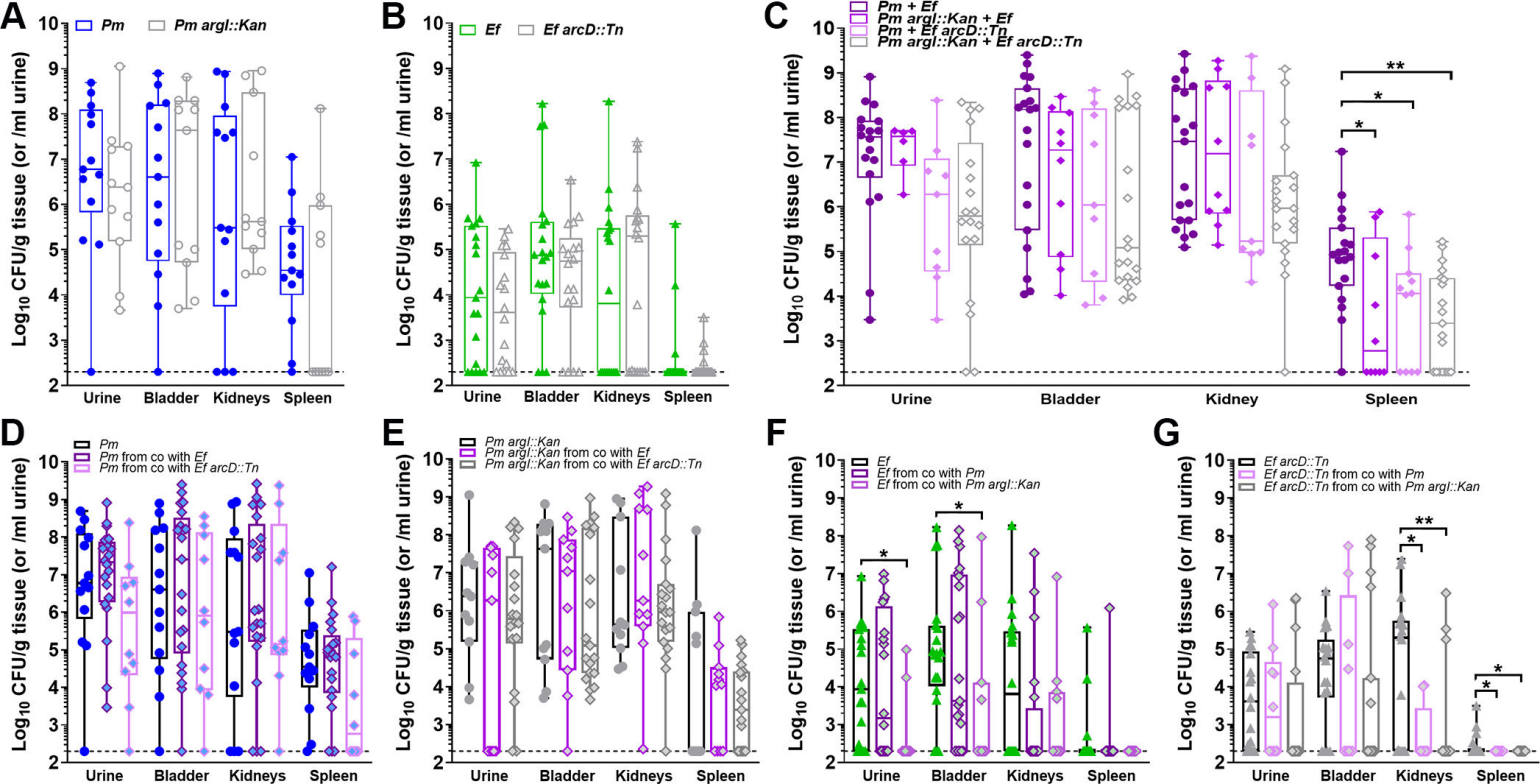
Metabolic interplay between *E. faecalis* and *P. mirabilis* contributes to secondary bacteremia. Bacterial CFUs in urine, bladder, kidneys, and spleen collected 96 hours post-infection in a CAUTI murine model. For single-species infections, animals were infected with 10^5^ CFUs of either wild-type *P. mirabilis* or *P. mirabilis argI::Kan* (**A**) or wild-type *E. faecalis* or *E. faecalis arcD::Tn* (**B**). For polymicrobial infections, mice were coinfected with a 50:50 mixture of the wild-type strains or their respective mutants (**C**). Each data point represents total CFUs per indicated organ. Data from three independent studies were analyzed via non-parametric Kruskal- Wallis one-way ANOVA. (**D–G**) The CFUs of each specific bacterial strain are displayed for single-species infection and each coinfection, with statistical significance determined by non-parametric Kruskal-Wallis one-way ANOVA comparison to CFUs from single-species infection, *P < 0.05 and **P < 0.01.

We previously demonstrated that polymicrobial infection with *E. faecalis* and *P. mirabilis* increases disease severity during experimental CAUTI compared to infection with either species alone, and we have also demonstrated the contribution of biofilm formation to bacterial pathogenesis in the context of CAUTI (38, 49). We therefore sought to determine the contribution of arginine/ornithine antiport and arginine biosynthesis to establishing polymicrobial infection and promoting dissemination to the kidneys and bloodstream in the well-established murine CAUTI model (31, 46, 50). Female CBA/J mice aged 6–8 weeks were transurethrally inoculated with 10^5^ CFUs of either wild-type *P. mirabilis*, *P. mirabilis argI::Kan*, wild-type *E. faecalis*, *E. faecalis arcD::Tn*, or polymicrobial combinations thereof, and a 4-mm silicone catheter segment was left in the bladder during inoculation. Mice were euthanized 96 hours post-inoculation, and bacterial burden was quantified in the urine, bladder, kidneys, and spleen as an indicator of bacteremia (Fig. 6).

During single-species infection, *P. mirabilis argI::Kan* colonized all organs of the urinary tract to a similar level as the wild type (Fig. 6A) and caused similar disease presentation but had a reduced capacity to disseminate to the bloodstream (Table 1). There were no differences in colonization (Fig. 6B) or infection severity (Table 1) for *E. faecalis arcD::Tn* compared with the wild type, indicating that arginine/ornithine antiport does not contribute to single-species infection. In the context of polymicrobial infection, both species were detected in all coinfected mice by differential plating (Fig. S6). There were no significant differences in total bacterial burden throughout the urinary tract (Fig. 6C), and the only difference in CFUs of each individual species across infection groups was a decrease in urine and bladder colonization by *E. faecalis* during coinfection with *P. mirabilis argI::Kan* (Fig. 6F) and in kidney and spleen colonization by *E. faecalis arcD::Tn* during coinfection with either *P. mirabilis* strain (Fig. 6G). Despite these very modest differences in the CFUs of a single constituent of the polymicrobial infection, mice coinfected with any of the mutant strains displayed reduced bacterial burden within the spleen (Fig. 6C). Strikingly, all polymicrobial infections involving the *E. faecalis arcD::Tn* mutant exhibited a significant reduction in the overall incidence of bacteremia as well as combined indicators of infection severity (kidney mottling and visible kidney stones; Table 1). Since no differences in bacterial burden or infection severity were observed during single-species infection with *E. faecalis arcD::Tn*, these data demonstrate that arginine/ornithine antiport by *E. faecalis* and metabolic interplay between *P. mirabilis* and *E. faecalis* contribute to virulence mechanisms that promote dissemination and bacteremia during polymicrobial infection.

**TABLE 1:** Disruption of metabolic cooperation between *E. faecalis* and *P. mirabilis* decreases severity of polymicrobial CAUTI^*a*^

Adverse health event or tissue abnormality	*Pm*	*argI::Kan*	*Ef*	*arcD::Tn*	*Pm* *+ Ef*	*Pm* *+ Ef arcD::Tn*	*Pm argI::Kan + Ef*	*Pm argI::Kan* *+ Ef arcD::Tn*
Bladder hematoma and/or blood in urine	1/13 (8%)	0/11 (0%)	0/20 (0%)	0/18 (0%)	0/19 (0%)	0/10 (0%)	0/9 (0%)	2/19 (10%)
Kidney hematoma	3/13 (23%)	0/11 (0%)	0/20 (0%)	0/18 (0%)	0/19 (0%)	0/10 (0%)	0/9 (0%)	0/19 (0%)
Kidney color change and/or mottling	1/13 (8%)	0/11 (0%)	0/20 (0%)	0/18 (0%)	5/19 (26%)	0/10 (0%)	1/9 (11%)	0/19 (0%)*
Kidney stone	2/13 (15%)	3/11 (27%)	0/20 (0%)	0/18 (0%)	6/19 (32%)	2/10 (20%)	2/9 (22%)	2/19 (10%)
Any abnormality	4/13 (31%)	3/11 (27%)	0/20 (0%)	0/18 (0%)	11/19 (58%)	2/10 (20%)*	3/9 (33%)	2/19 (10%)**
Bacteremia	12/13 (92%)	5/11 (45%)*	3/20 (15%)	4/18 (22%)	18/19 (95%)	5/10 (50%)**	7/9 (78%)	12/19 (63%)*

^*a*^ Presence of tissue abnormalities during murine CAUTI was determined by gross macroscopic inspection of organs, and presence of bacteremia was defined as having >200 CFU/mL of bacteria in the spleen. *P < 0.05 and **P < 0.01 by Fisher’s exact test; single-species infections with each mutant were compared with those of the respective parental strain, while polymicrobial infections were compared with those of the wild-type *P. mirabilis* + wild-type *E. faecalis* infection group.

## DISCUSSION

**Fig 7:**
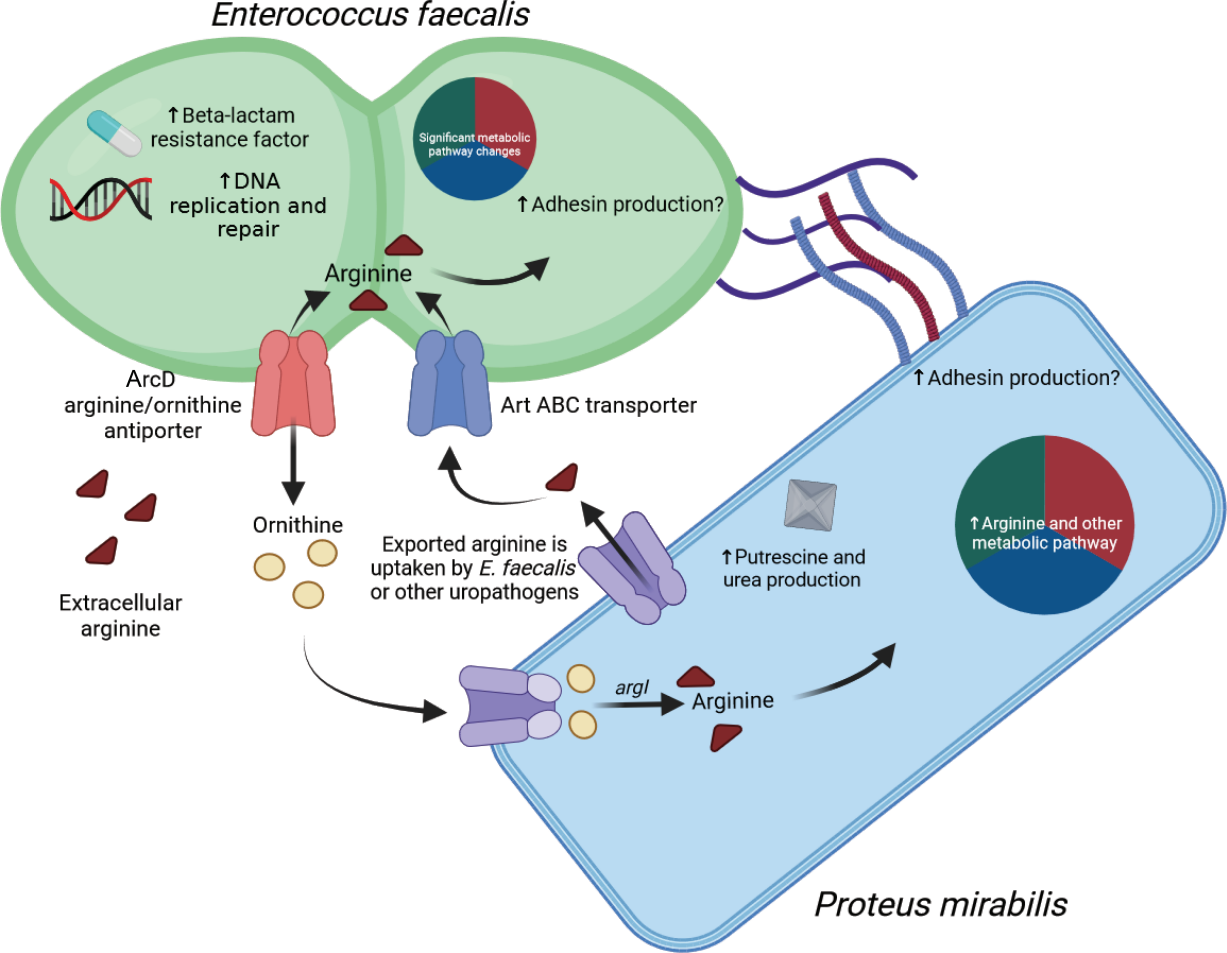
Working model of how metabolic interplay between *E. faecalis* and *P. mirabilis* facilitates biofilm enhancement. *E. faecalis* imports arginine and secretes ornithine via the ArcD antiporter. *P. mirabilis* imports the now-abundant ornithine and generates arginine through the sequential action of ArgI, ArgG, and ArgH. The resulting arginine has three possible fates: (i) catabolism to the polyamine putrescine, consequently resulting in production of urea; (ii) incorporation into proteins, potentially including those involved in enhanced biomass production; and (iii) export out of the cell, which would provide additional arginine for import by *E. faecalis*. Notably, *E. faecalis* has additional arginine import mechanisms that are not linked to ornithine antiport, such as the Art ABC transporter. This metabolic interplay ultimately increases the total protein content of the polymicrobial biofilm and is associated with increased abundance of potential adhesins in both species.

Bacterial biofilms have long been noted to be vital for pathogenesis and disease progression in a variety of contexts, including CAUTI (51–54). There is growing appreciation for the fact that many diseases are polymicrobial, where the network of interactions between bacterial species and the host are important determinants of the overall course of disease development (10, 31, 54–56). However, there remains a paucity of studies addressing the interactions that contribute to polymicrobial biofilm formation, colonization, and pathogenesis. Previously, we demonstrated that *E. faecalis* and *P. mirabilis* are frequent co-colonizers in catheterized patient populations and they co-localize to form unique biofilm communities with enhanced biofilm biomass, persistence, and antibiotic resistance (10). Herein, we demonstrate that arginine/ornithine antiport by *E. faecalis* feeds into L-arginine biosynthesis and metabolism by *P. mirabilis*, resulting in a contact-dependent increase in protein content and biofilm biomass compared with single-species biofilms that we hypothesize is due to coordinated production or activity of adhesins in each species (Fig. 7). We further demonstrate that metabolic interplay influences biofilm formation *in vitro* including under conditions that mimic catheter urine flow without altering bacterial viability under any condition, and this interplay also contributes to mediating disease severity in a mouse model of polymicrobial CAUTI, particularly the development of bacteremia.

This study adds to a growing body of work demonstrating that metabolic cross-feeding is a determinant of polymicrobial infections. Keogh et al. (40) demonstrated that L-ornithine secretion from *E. faecalis* increased *E. coli* siderophore production and biofilm growth under iron limitation, as well as persistence in a wound infection model (40). More recently, work by Smith et al. (39) demonstrated that *E. faecalis* enhances the fitness and virulence of the gut pathogen *Clostridioides difficile* by providing a source of fermentable amino acids, including ornithine (39). In both of these studies, L-ornithine from *E. faecalis* enhanced growth of the partner species. In contrast, our work demonstrates a growth-independent role for L-ornithine in mediating contact-dependent polymicrobial interactions. Together, these studies underscore the pivotal role that *E. faecalis* arginine/ornithine antiport plays in mediating different polymicrobial interactions in multiple disease contexts and further highlights the potential of L-ornithine as a common metabolite cue.

Considering that supplementation with either ornithine, arginine, or agmatine restored polymicrobial biofilm enhancement for the mutant strains while putrescine did not, our data indicate that either intracellular L-arginine stores or biosynthesis intermediates are likely the key mediators of biofilm enhancement in *P. mirabilis*. Our prior studies have demonstrated an important contribution of L-arginine to *P. mirabilis* fitness and virulence; specifically, L-arginine acts as an environmental cue to promote swarming motility, and catabolism to agmatine via SpeA contributes to acid tolerance and motility by influencing membrane potential in addition to fueling putrescine biosynthesis (41). In our prior genome-wide transposon insertion site sequencing (Tn-seq) study, we also found that polymicrobial infection with another common coinfection partner, *Providencia stuartii*, causes *P. mirabilis* to require the L-arginine biosynthetic pathway (including *argI*) for optimal fitness (47). Combined with the current study, these observations hint at the potential for L-arginine to act as a key determinant of *P. mirabilis* virulence and fitness in polymicrobial CAUTI.

The observation that ornithine supplementation restored enhancement of biofilms formed by co-culture of *P. mirabilis argI::Kan* with *E. faecalis arcD::Tn* was initially surprising since disrupting ornithine carbamoyltransferase should prevent generation of citrulline and subsequently arginine by *P. mirabilis*. However, it is possible that ornithine catabolism via *speF* may at least partially compensate for loss of *argI* under these conditions. Unfortunately, testing this hypothesis requires an *argI/speF* double mutant in *P. mirabilis* for which numerous attempts proved unsuccessful, suggesting that ornithine catabolism by at least one of these pathways is required for *P. mirabilis* viability *in vitro*.

While the specific fate of arginine during polymicrobial infection remains to be determined, we hypothesize that both species may utilize the excess L-arginine generated from L-ornithine for production of specific proteins that ultimately establish the protein-mediated biofilm enhancement and possibly tissue attachment and invasion during infection. Our proteomics experiments revealed two potential *P. mirabilis* adhesins that were enriched in polymicrobial biofilms: a putative multifunctional-autoprocessing repeats-in-toxin (RTX) protein and two components involved in the production of a putative chaperone-usher fimbria (the Fim5D fimbrial protein and associated Fim5C chaperone protein). RTX proteins are part of a family of pore-forming cytolysins produced mainly by Gram-negative bacteria, and they can have diverse functions including playing a role in biofilm formation (57–59). For example, the most well-characterized RTX adhesin is the *Pseudomonas fluorescens* protein LapA, which was shown to be essential for biofilm formation (59, 60). Fimbriae are also known to play a vital role in *P. mirabilis* biofilm formation and mediate adherence to the catheter surface (32, 61), although the specific contribution of Fim5 has yet to be explored. Interestingly, the putative RTX protein and several genes within the *fim5* operon were previously identified as *P. mirabilis* fitness factors during polymicrobial CAUTI with *P. stuartii* but not during single-species infection, suggesting that both may play a specific role in mediating polymicrobial interactions (47). Our proteomics experiments also revealed three enriched *E. faecalis* proteins that have previously been associated with biofilm formation and may contribute to polymicrobial biofilm enhancement: the endocarditis and biofilm-associated pilus tip protein EbpA, an ankyrin repeat family protein, and a methyl-accepting chemotaxis family protein (62, 63). The contribution of each of these putative *P. mirabilis* and *E. faecalis* adhesins to polymicrobial interactions is an active area of ongoing investigation.

Metabolomics analysis of single and polymicrobial biofilms revealed the full complexity of interactions between just two bacterial species and the large influence that culture media have on these processes. While it was exciting that arginine and ornithine followed clearly expected trends based on our hypotheses, several other amino acids warrant further investigation. For example, the accumulation of tyrosine in polymicrobial biofilms involving wild-type *E. faecalis* that is absent or reduced when *arcD* is disrupted suggests that cross-feeding promotes tyrosine biosynthesis. Since *E. faecalis* does not encode the necessary genes for tyrosine biosynthesis, arginine/ornithine antiport by *E. faecalis* appears to be stimulating tyrosine production by *P. mirabilis*. Tyrosine can be converted into a variety of secondary metabolites, such as fumarate or pyruvate, and there is evidence of host-pathogen cross-talk mediated by tyrosine (64–66). We also observed an increase in GABA in polymicrobial biofilms only when arginine/ornithine antiport was disrupted in *E. faecalis*. Notably, this only occurred in TSB-G and was lost during culture in human urine conditions, highlighting the importance of the nutrient environment. *E. faecalis* again lacks the pathway for production of GABA, and *P. mirabilis* encodes a glutamate/GABA antiporter. Since glutamate is no longer depleted during co-culture of *P. mirabilis* with *arcD::Tn*, these combined observations suggest that disruption of arginine/ornithine antiport in *E. faecalis* likely alters glutamate metabolism in *P. mirabilis*, resulting in the accumulation of GABA in the supernatant. Together, our data highlight the multifaceted impact of altering the function of a single amino acid transporter.

A caveat for our *in vitro* biofilm data and murine CAUTI infection data is that *in vivo* biofilm formation on a catheter surface was not quantified. While it is expected that the *in vitro* biofilm defect would be replicated on the catheter segment during experimental infection, the presence of host proteins and immune factors could alter polymicrobial biofilm interactions and resulting biomass. Further investigation into the composition and architecture of the *in vivo* catheter biofilm will provide insight into bacteria-host dynamics that might influence biofilm biomass and progression from catheter colonization to disseminated infection.

Given that *P. mirabilis* and *E. faecalis* both exhibit intrinsic resistance to several antibiotics and that polymicrobial biofilm formation enhances resistance, further examination of the metabolic interplay between these and other species could shed light on new approaches to prevent or disrupt formation of highly resistant polymicrobial biofilms. Our data suggest that targeting ornithine metabolism and arginine biosynthesis may represent a new avenue for exploration, especially as disrupting these pathways also decreased the risk of developing severe disease during polymicrobial infection without substantial risk of developing resistance as neither pathway was required for viability of either species. However, it is important to note that the bladder environment typically includes two to five distinct bacterial species during long-term catheterization (39, 67–69). The addition of other uropathogens is likely to modulate or influence cross-feeding interactions described here through a variety of mechanisms that change the nutrient landscape, spatial structure of the community, or community metabolism (70, 71). Additionally, other uropathogens such as *P. stuartii*, *E. coli*, and *Morganella morganii* have been shown to modulate virulence factor production and activity in *P. mirabilis* and may also contribute to biofilm formation (38, 72). Thus, the impact of other common uropathogens on L-ornithine-driven biofilm enhancement warrants further exploration, particularly if other species can disrupt this interaction though competition for ornithine, arginine, or other metabolites.

## MATERIALS AND METHODS

### Bacterial strains

*P. mirabilis* strain HI4320 was isolated from the urine of a long-term catheterized patient in a chronic care facility (73). All *P. mirabilis* mutants used in this study were generated by inserting a kanamycin resistance cassette into the gene of interest following the Sigma TargeTron group II intron protocol as previously described (74). The *speF*, *speA*, and *speB* mutants were previously constructed (41), while the *argI* mutant was generated for this study. Mutants were verified by selection on kanamycin and PCR. The complemented *argI* strain was generated by cloning *argI* with ~500-bp flanking regions into pGen-Amp and verified by PCR. The *E, faecalis* strain used in this study is OG1RF (75, 76), for which the *arcD::Tn* mutant was previously generated via mariner transposon mutagenesis (40, 42, 43).

### Whole genome sequencing and SNV analysis of the *E. faecalis arcD::Tn* mutant

Genomic DNA was extracted from *E. faecalis* OG1RF and the *E. faecalis arcD::Tn* mutant using the DNeasy Blood and Tissue Kit (Qiagen, Germantown, Maryland, USA) according to the manufacturer’s instructions and sent to SeqCoast Genomics for whole genome sequencing. Samples were prepared using the Illumina DNA Prep Tagmentation Kit and IDT For Illumina Unique Dual Indexes. Sequencing was performed on the Illumina NextSeq2000 platform using a 300 cycle flow cell kit to produce 2 × 150 bp paired reads. One to two percent PhiX control was spiked into the run to support optimal base calling. Read trimming and run analytics were performed using Trimmomatic v0.39.0 (77). The processed reads were assembled using spades v3.15.5 wrapped in Unicycler v0.5.0 using the complete *E. faecalis* OG1RF genome as a reference to fill in the gaps created by short-read *de-novo* assembly (78–80). Read mapping was done using Bowtie2 and SAMtools (wrapped in Unicycler) (81, 82). The complete genome assemblies that passed quality filtering were annotated using prokka v1.14.5 (83). Gene presence and absence were assessed using prokka-annotated assemblies in gff3 format in roary v3.13.0 using a core threshold cluster of 95% (84). To determine if any SNV occurred in the *E. faecalis arcD::Tn* strain, reads were mapped against the reference complete genome of *E. faecalis* OG1RF (published in 2011) using breseq v0.36.1 (80, 81, 85). The genome sequences are available under BioProject PRJNA1165210 (*Enterococcus faecalis* OG1RF and *arcD::Tn* genome sequencing).

### Bacterial culture conditions

*P. mirabilis* was cultured at 37°C with shaking at 225 RPM in 5 mL of low-salt LB broth (10 g/L tryptone, 5 g/L yeast extract, and 0.1 g/L NaCl) or on plates solidified with 1.5% agar. *E. faecalis* was cultured in 5 mL of brain heart infusion (BHI) broth at 37°C with shaking at 225 RPM or on BHI solidified with 1.5% agar. Both species were also cultured in TSB-G. Mutant strains were cultured with 50 µg/mL kanamycin (*P. mirabilis*) or 8 µg/mL chloramphenicol (*E. faecalis*). Filter-sterilized pooled human urine from at least 20 de-identified female donors was purchased from Cone Bioproducts (Sequin, TX) stored at −20C until use.

### Crystal violet staining of bacterial biofilms

Overnight cultures were adjusted to ~10^7^ CFU/mL in TSB-G or pooled human urine, and 750 µL was dispensed in triplicate into the wells of tissue culture-treated 24-well plates (Falcon 353047). For polymicrobial biofilms, 375 µL of each strain was added. Sterile media served as a blank for background subtraction. Biofilms were incubated for 24 hours at 37°C in partially sealed bags with a damp paper towel, supernatants were gently aspirated, and biofilms were washed twice with 1 mL phosphate buffered saline (PBS). Biofilms were ethanol fixed, stained with crystal violet, and analyzed as described previously (10). Crystal violet absorbance in all figures is expressed relative to absorbance in the *E. faecalis* monoculture biofilm wells.

### Biofilm viability

Biofilms were established as above, supernatants were aspirated, and biofilms were gently washed with 1 mL of sterile PBS and resuspended in PBS. Suspensions were diluted and plated onto low-salt LB (*P. mirabilis* CFUs) and BHI with 150 µg/mL streptomycin (*E. faecalis* CFUs) using an EddyJet 2 spiral plater (Neutec Group) for determination of CFUs using a ProtoCOL 3 automated colony counter (Synbiosis).

### Biofilm compositional analysis

Biofilms were established as above, and compositional analysis was performed as previously described (49). Briefly, supernatants were aspirated and all replicate biofilms from the same inoculum were resuspended in 3 mL of sterile Milli-Q water. Extracellular DNA (eDNA) was determined using the PicoGreen assay (Invitrogen, MP07581), carbohydrate using the Total Carbohydrate Assay Kit (Sigma, Cat# MAK104-1KT), and protein via Pierce BCA Protein Assay Kit (Thermo Fisher, Cat# 23250).

### Liquid-chromatography mass spectrometry analysis of bacterial biofilms

Sample preparation and data analysis are described in detail in the Supplemental Methods. The mass spectrometry proteomics data have been deposited to the ProteomeXchange Consortium via the PRIDE partner repository with the data set identifier PXD041693 (86). Pathway analysis was performed on each list of differentially abundant proteins in the polymicrobial biofilms compared with single-species biofilms. *P. mirabilis* and OG1RF identifiers in the data sets were mapped to KEGG pathways using the R package KEGGREST (87).

### Metabolomics analysis of bacterial biofilm supernatant

Biofilms were established as above and supernatants were collected at 2, 6, or 24 hours, filtered, and stored at −80°C until use. Amino acids were quantified as previously described using a Waters Acquity uPLC System with an AccQ-Tag Ultra C18 1.7 µm 2.1 × 100 mm column and a Photodiode Detector Array (88). Supernatants were thawed, centrifuged at 13,000 × *g* for 5 minutes, and amino acids were derivatized using the Waters AccQ-Tag Ultra Amino Acid Derivatization Kit (Waters Corporation, Milford, MA) and analyzed using the UPLC AAA H-Class Application Kit (Waters Corporation, Milford, MA) according to manufacturer’s instructions. Agmatine was purchased from Sigma-Aldrich (A7127) and was diluted in mass spectrometry grade methanol. Blanks and standards were run every eight samples. All chemicals and reagents used were mass spectrometry grade.

### Glass bladder model

A previously described glass bladder model was used, in which a 500mL water-jacketed glass vessel is maintained at 37°C via a circulating water bath, a Foley catheter is inserted and inflated, and AUM is supplied into the bladder at a constant flow rate (0.5–1.0mL/min) through a peristaltic pump (89, 90). Bladders were inoculated with 10^8^ CFUs of *P. mirabilis*, *E. faecalis*, or a 1:1 mixture; samples were collected from the catheter port at 0, 3, 6, 9, 12, and 24 hours to track bacterial CFUs; and catheter segments were processed for CFUs and crystal violet staining at 24 hours. Full details are provided in the Supplemental Methods.

### Mouse model of CAUTI

Female CBA/J mice aged 6–8 weeks (Jackson Laboratory) were anesthetized with a weight-appropriate dose of ketamine/xylazine (80–120 mg/kg ketamine and 5–10 mg/kg xylazine) via IP injection and transurethrally inoculated with 50 µL of *P. mirabilis*, *E. faecalis*, each mutant, or a 1:1 mixture (1 × 10^5^ CFU/mouse). A 4-mm segment of sterile silicone tubing (0.64 mm O.D., 0.30 mm I.D., Braintree Scientific Inc.) was advanced into the bladder during inoculation to be retained for the duration of the study described previously (38, 91). Bacterial burden was quantified in the urine, bladder, and kidneys as well as the spleen as an indicator of bacteremia. Full details are provided in the Supplemental Methods.

### Statistical analysis

All analyses were performed using GraphPad Prism, version 9.3 (GraphPad Software) with a 95% CI.

## SUPPLEMENTAL MATERIAL

Supplemental figures mbio.02164-24-s0001.pdf Figures S1-S6.

Supplemental text mbio.02164-24-s0002.pdf Supplemental methods.

Table S1 mbio.02164-24-s0003.xlsx Proteomics full data set.

Table S2 mbio.02164-24-s0004.xlsx Comparison of *arcD*::Tn and wt OG1RF genome sequences.

Table S3 mbio.02164-24-s0005.xlsx Metabolomics full data set with statistical analyses.

ASM does not own the copyrights to Supplemental Material that may be linked to, or accessed through, an article. The authors have granted ASM a non-exclusive, world-wide license to publish the Supplemental Material files. Please contact the corresponding author directly for reuse.
