# Supplementary material for: Metabolic interplay between Proteus mirabilis and Enterococcus faecalis facilitates polymicrobial biofilm formation and invasive disease
Source: mBio. 2024 Oct 30;15(12):e02164-24. doi: 10.1128/mbio.02164-24 (PMC11640290; doi:10.1128/mbio.02164-24)
Supplement: Supplemental figures — Figures S1-S6. [file mbio.02164-24-s0001.pdf]

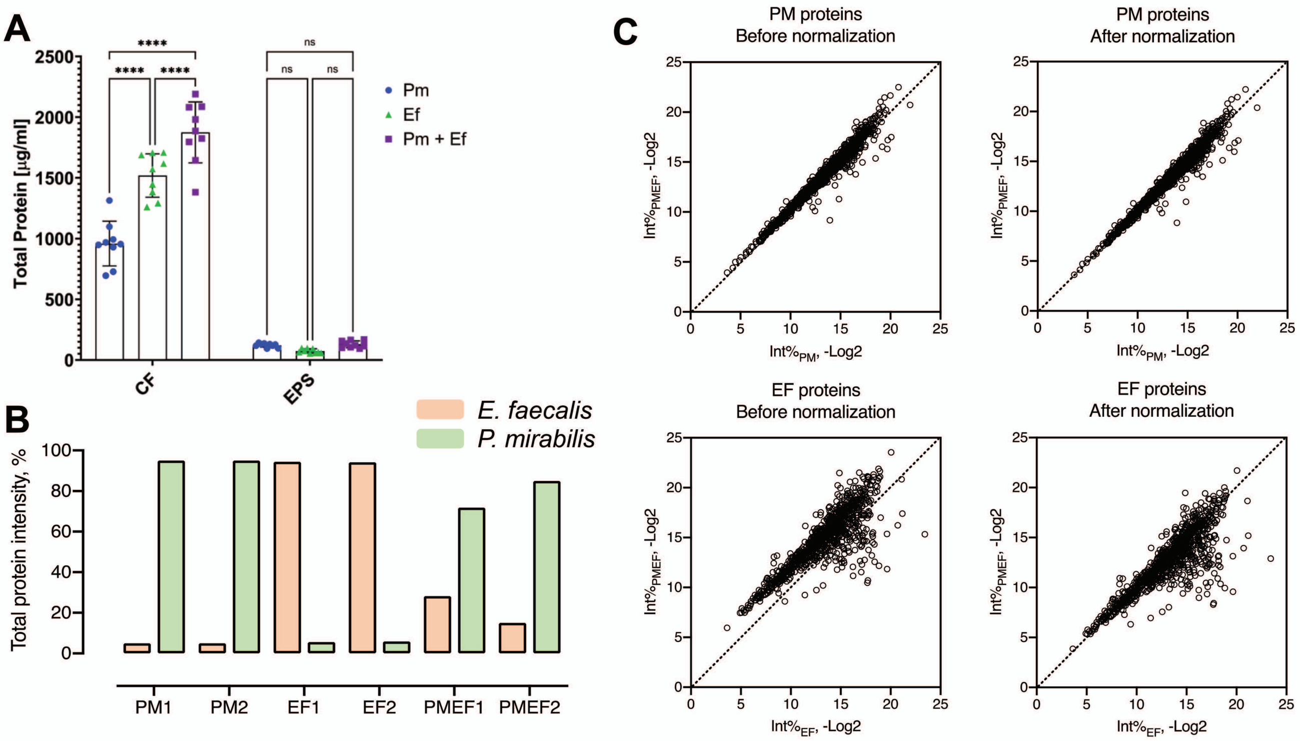

Supplemental Figure 1. Characterization of protein enrichment in polymicrobial biofilms. (A) Biofilm fractionation experiments were performed to separate the cell-associated fraction (CF) from the soluble extrapolymeric substance (EPS) and protein levels were quantified by BCA. Data represent the mean ± SD for three independent experiments with at three replicates each. ns = non-significant, **** = P<.0001 by one-way ANOVA. (B) Protein quantification was performed by UHR-IonStar. Peptides shared between the two species (<1% of all peptides quantified) were removed from quantification. Protein intensities were converted to Protein intensity percentage to compensate for inter-species biases. Biological replicates are represented by "1" and "2" for each biofilm type. The total protein intensity percentages were quite consistent in PM- and EF-only samples (~95%), while in the PMEF samples, the percentages are largely different. As this would bias the comparison of protein abundance in PM-/EF-only samples and PMEF samples, an additional normalization step was performed for each protein, the results of which are displayed in panel C:

Int%PM in PMEF, adj = Int%PM in PMEF * Int%PM in PM, total / Int%PM in PMEF, total

Int%EF in PMEF, adj = Int%EF in PMEF * Int%EF in EF, total / Int%EF in PMEF, total

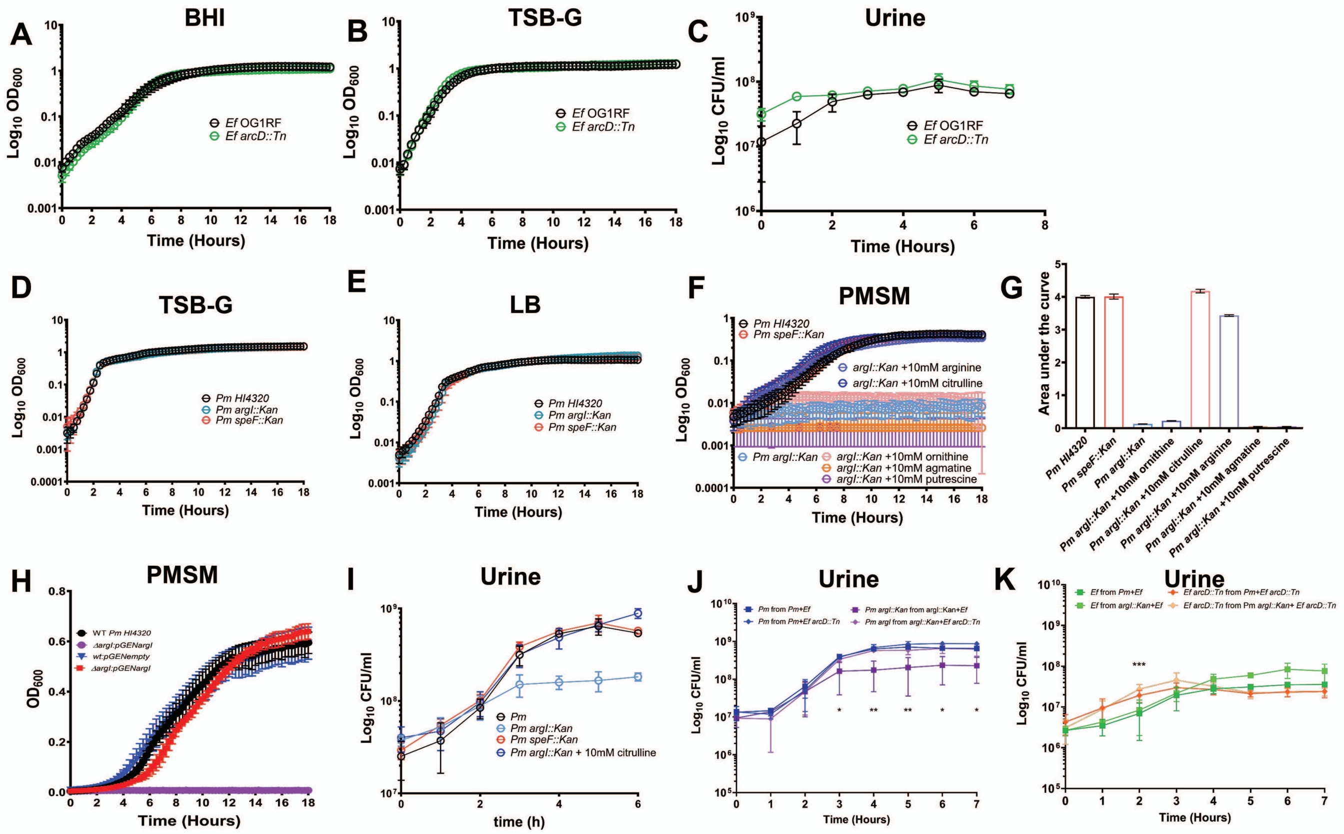

Supplemental 2. Arginine biosynthesis from ornithine is a key determinant of *P. mirabilis* growth under nutrient limitation. (A-C) *E. faecalis* growth curves in A) BHI, B) TSB-G, and C) pooled human urine. (D-G) *P. mirabilis* growth curves in D) TSB-G E) LB, F and G) PMSM (*Proteus* minimal salts media) without supplementation or supplemented with 10 mM ornithine, 10mM arginine, 10 mM citrulline, 10 mM agmatine, or 10 mM putrescine. Panel G displays area under the curve values from Panel F. (H) Growth curve of complemented *argF* mutant in PMSM. (I) CFUs of *P. mirabilis* and mutants during growth in pooled human urine. (J-K) CFUs of *P. mirabilis* strains (J) or *E. faecalis* strains (K) during co-culture in pooled human urine. * $p < 0.05$, ** $p < 0.01$, *** $p < 0.001$ by two-way ANOVA. Data represent mean ± SD of at least three independent experiments with at least three replicates each.

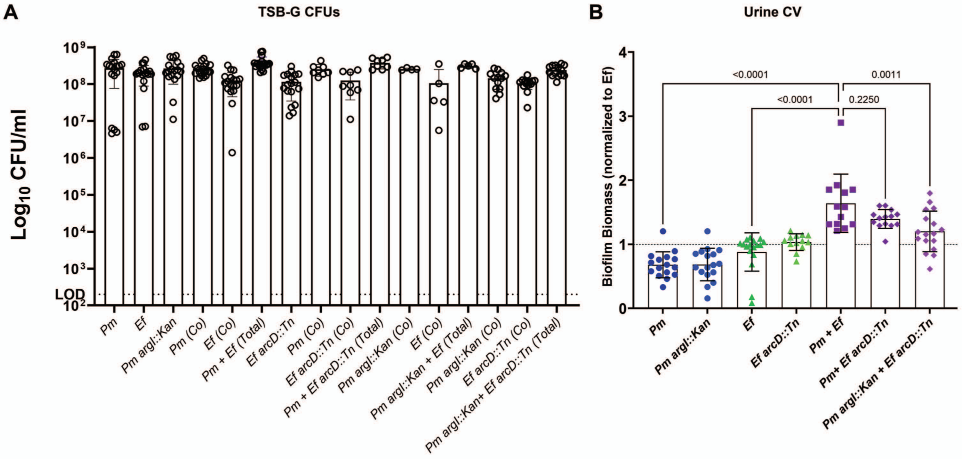

Supplemental 3. Enhanced biomass of polymicrobial biofilms is not driven by changes in viability in TSB-G and requires both arginine/ornithine antiport and arginine biosynthesis in human urine. (A) CFUs of biofilms grown for 24-hours in TSB-G. Data represent the mean ± SD for at least three independent experiments with at least two replicates each. (B) Crystal violet staining of single or polymicrobial biofilms grown for 24 hours in pooled human urine. Data represent the mean ± SD for 3-5 independent experiments with at least two replicates each.

	Ef WT			Ef *arcD*			Pm WT			Pm *argI*			Pm *speA*		
	2 Hr	6 Hr	24 Hr	2 Hr	6 Hr	24 Hr	2 Hr	6 Hr	24 Hr	2 Hr	6 Hr	24 Hr	2 Hr	6 Hr	24 Hr
Agmatine	-0.095	0.281	0.348	1.460	0.552	0.319	-1.014	0.621	-0.051	0.714	0.600	-0.185	0.701	0.373	1.189
Arginine	-0.029	-3.968	-3.865	-2.871	-3.045	-0.475	-1.074	-2.115	-4.920	-0.589	-2.067	-3.531	-0.595	-1.820	-3.306
Ornithine	2.092	4.540	4.549	-0.162	-0.364	-0.186	-0.727	-0.615	-1.429	-0.380	-0.359	-1.653	-0.248	-1.597	-1.422
Valine	0.129	-0.305	-0.318	-0.070	-0.113	-0.363	-0.547	0.061	-1.132	-0.226	-0.089	-0.484	-0.283	-0.286	-0.426
Isoleucine	0.114	-0.347	-0.372	-0.082	-0.168	-0.401	-0.558	-0.013	-3.622	-0.237	-0.128	-0.926	-0.289	-0.337	-0.865
Leucine	0.116	-0.381	-0.431	-0.092	-0.207	-0.471	-0.650	-0.592	-6.000	-0.315	-0.793	-3.395	-0.344	-0.700	-2.774
Phenylalanine	0.055	-0.483	-0.671	-0.166	-0.407	-0.693	-0.563	-0.211	-6.000	-0.293	-0.324	-1.656	-0.377	-0.471	-1.530
Tryptophan	-0.094	-0.510	-0.797	-0.356	-0.597	-0.643	-0.547	-0.165	-3.457	-0.277	-0.266	-1.498	-0.371	-0.584	-1.550
Lysine	0.135	-0.335	-0.315	-0.056	-0.056	-0.388	-0.659	0.157	-4.555	-0.227	-0.049	-0.533	-0.222	-0.194	-0.357
Methionine	0.062	-0.377	-0.456	-0.148	-0.252	-0.518	-0.560	-0.033	-6.000	-0.292	-0.169	-2.456	-0.352	-0.367	-1.640
Tyrosine	-0.354	-3.673	-3.695	-0.559	-3.663	-3.860	-0.511	-0.065	0.794	-0.309	-0.261	0.330	-0.449	-0.855	-1.203
Aspartic Acid	0.176	0.010	0.149	0.050	0.291	0.113	-1.609	-4.527	-5.151	-1.093	-4.660	-5.096	-0.401	-4.795	-4.982
Glutamic Acid	0.121	-0.101	-0.036	0.001	0.176	-0.045	-0.612	0.038	-6.000	-0.157	-0.160	-6.000	-0.164	-0.221	-6.000
Threonine	0.119	-0.268	-0.262	-0.034	-0.049	-0.287	-0.543	0.114	-1.692	-0.181	-0.044	-1.818	-0.235	-0.198	-1.449
Alanine	0.171	-0.167	-0.072	0.009	0.055	-0.201	-0.551	0.282	-0.600	-0.148	0.123	-0.159	-0.183	-0.036	0.017
GABA	0.467	-0.212	-0.878	0.301	0.068	-0.336	-0.583	-0.181	-0.500	-0.178	0.017	-1.382	-0.178	-0.352	-1.350
Proline	0.188	-0.173	-0.192	-0.030	-0.027	-0.261	-0.442	0.356	-2.613	-0.130	0.206	-2.111	-0.210	-0.050	-0.832
Hyroxylysine	0.101	-1.141	-1.166	-0.135	-1.313	-1.253	-0.509	-0.373	-0.952	-0.547	-0.326	-0.237	-0.131	-1.234	-0.281
AABA	0.053	-0.617	-0.661	-0.332	-0.767	-1.293	-0.521	0.495	-0.452	-0.266	0.241	1.296	-0.201	-0.358	0.656
Cysteine	-0.043	-0.809	-0.823	-0.611	-0.594	-0.939	-1.108	0.140	-0.781	-0.636	-0.900	-0.884	-0.368	-1.164	-1.143
Histidine	0.086	-0.295	-0.313	-0.091	-0.215	-0.428	-0.609	-0.542	-6.000	-0.226	-0.609	-1.811	-0.320	-0.631	-1.929
Asparagine	0.085	-0.168	-0.170	-0.060	-0.113	-0.329	-2.086	-6.000	-6.000	-1.769	-5.785	-3.861	-1.076	-5.672	-3.668
Taurine	0.018	-0.623	-0.396	-0.152	-0.521	-0.793	-0.890	-1.228	-2.974	-0.201	-0.808	-2.641	-0.474	-0.772	-2.595
Serine	-2.287	-4.740	-2.165	-1.604	-2.796	-4.410	-5.836	-5.646	-3.206	-5.333	-5.629	-4.286	-3.952	-5.978	-5.783
Glutamine	-0.090	-0.922	-0.386	2.674	2.297	-0.450	-3.215	-5.024	-5.024	-5.024	-5.024	-5.024	-5.024	-5.024	-5.024

	Pm + Ef			Pm + *arcD*			*argI* + Ef			*argI* + *arcD*			*speA* + Ef			*speA* + *arcD*		
	2 Hr	6 Hr	24 Hr	2 Hr	6 Hr	24 Hr	2 Hr	6 Hr	24 Hr	2 Hr	6 Hr	24 Hr	2 Hr	6 Hr	24 Hr	2 Hr	6 Hr	24 Hr
Agmatine	0.574	0.408	0.137	-0.157	1.395	0.343	-1.014	1.235	-0.004	0.720	0.499	-1.014	-0.157	-1.014	-1.014	-1.014	-1.014	-1.014
Arginine	-0.424	-3.872	-5.938	-0.594	-3.275	-1.858	-0.417	-3.846	-4.455	-0.125	-1.269	-2.007	-0.051	-3.661	-4.104	-0.235	-0.924	-1.595
Ornithine	1.182	-1.802	-0.720	-0.814	-1.490	-1.302	1.213	-1.644	0.885	-0.230	-1.570	-1.481	1.470	-1.911	-1.243	-0.576	-0.821	-2.063
Valine	-0.121	-0.365	-0.477	-0.356	0.050	-0.084	-0.109	-0.035	-0.359	0.098	-0.287	-0.226	0.154	-0.353	0.034	-0.058	-0.257	-0.437
Isoleucine	-0.132	-0.450	-3.322	-0.367	0.025	-0.163	-0.127	-0.090	-0.759	0.092	-0.338	-0.303	0.161	-0.411	-0.270	-0.065	-0.310	-0.498
Leucine	-0.172	-0.884	-6.000	-0.404	-0.286	-0.619	-0.168	-0.546	-3.666	0.048	-0.671	-0.779	0.137	-0.693	-2.203	-0.092	-0.503	-0.744
Phenylalanine	-0.171	-0.737	-6.000	-0.344	-0.099	-0.649	-0.265	-0.230	-1.795	0.049	-0.563	-0.769	0.208	-0.489	-0.878	-0.149	-0.428	-0.936
Tryptophan	-0.167	-0.636	-3.316	-0.340	-0.018	-0.222	-0.278	-0.151	-1.308	0.033	-0.470	-0.373	0.194	-0.404	-0.462	-0.168	-0.345	-0.594
Lysine	-0.131	-0.223	-1.311	-0.494	0.001	-0.036		-0.117	-0.472	0.076	-0.189	-0.195	0.016	-0.423	-0.145	-0.029	-0.244	-0.383
Methionine	-0.178	-0.563	-5.870	-0.376	-0.020	-0.236	-0.212	-0.150	-1.592	0.041	-0.428	-0.390	0.179	-0.443	-0.788	-0.126	-0.322	-0.578
Tyrosine	-0.667	1.431	2.357	-0.978	-1.658	-1.279	-0.725	1.780	2.129	-0.539	-1.924	-1.491	-0.227	1.593	2.273	-0.679	-3.381	-2.876
Aspartic Acid	-0.473	-4.906	-4.953	-0.934	-3.672	-4.415	-0.421	-4.369	-5.335	-0.290	-4.042	-4.545	-0.085	-4.759	-4.679	-1.997	-3.805	-4.177
Glutamic Acid	-0.048	-0.084	-6.000	-0.410	0.096	-0.313	0.045	-0.032	-4.010	0.094	-0.032	-0.429	0.033	-0.194	-2.107	0.005	-0.002	-0.323
Threonine	-0.083	-0.196	-0.649	-0.340	0.173	0.067	-0.056	0.076	-0.714	0.116	-0.127	-0.053	0.130	-0.205	-0.315	-0.032	-0.117	-0.267
Alanine	-0.042	-0.099	-0.117	-0.327	0.175	0.170	-0.004	0.112	-0.122	0.133	-0.087	0.044	0.123	-0.191	0.233	0.020	-0.087	-0.191
GABA	-0.031	-0.308	-0.346	-0.495	1.507	3.456	-0.141	-0.205	-1.770	0.367	1.139	3.236	0.337	-0.284	-0.549	0.277	0.866	2.308
Proline	-0.049	-0.058	0.490	-0.255	0.341	0.298	-0.060	0.329	0.439	0.186	-0.034	0.170	0.237	-0.038	0.835		-0.052	-0.166
Hyroxylysine	-0.231	-1.053	-1.046	-0.287	-1.081	-1.232	-0.154	-1.223	-0.316	-0.031	-0.854	-1.371	0.052	-1.000	-0.091	-0.170	-0.794	-1.334
AABA	-0.169	-0.483	-0.332	-0.622	-0.480	-0.586	-0.095	-0.604	0.499	-0.127	-0.720	-0.708	0.076	-0.810	0.786	-0.227	-0.871	-0.758
Cysteine	-0.641	-1.239	0.307	-1.041	-1.050	-1.033	-0.524	-1.276	-1.249	-0.510	-1.196	-0.901	-0.257	-0.988	-1.791	-0.590	-0.928	-1.132
Histidine	-0.141	-0.939	-6.000	-0.361	-0.107	-0.577	-0.183	-0.512	-2.093	0.103	-0.514	-0.710	0.201	-0.611	-1.398	-0.149	-0.415	-0.778
Asparagine	-0.523	-5.830	-6.000	-0.826	-4.790	-2.964	-0.556	-5.495	-6.000	-0.353	-4.914	-5.772	-0.208	-5.401	-6.000	-0.430	-5.285	-5.509
Taurine	-0.297	-2.599	-4.326	-0.758	-0.324	-0.719	-0.349	-2.270	-2.649	0.146	-0.553	-0.805	0.260	-0.740	-0.825	-0.521	-0.979	-2.464
Serine	-4.763	-5.913	-4.651	-4.721	-5.472	-4.915	-4.343	-6.000	-4.447	-4.232	-5.597	-3.730	-3.150	-5.927	-4.017	-3.135	-5.692	-3.069
Glutamine	-1.752	-5.024	-2.711	-3.044	-0.263	-5.024	-1.845	-5.024	-5.024	-0.972	-5.024	-5.024	-0.649	-5.024	-5.024	-1.213	-5.024	-5.024

Supplemental 4. Expanded metabolomics analysis of supernatants collected from single and polymicrobial *P. mirabilis* and *E. faecalis* biofilms to determine the impact of *P. mirabilis* arginine biosynthesis and metabolism on metabolite profiles at 2, 6, and 24 hours post inoculation in TSB-G. Average metabolite concentrations from two independent experiments are represented as log2 fold change compared to an uninoculated media alone control (either TSB-G or urine) collected at the 2 hour time point.

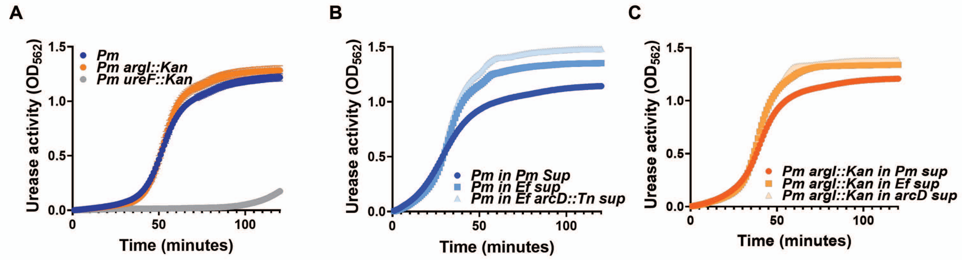

Supplemental 5. Contribution of arginine/ornithine transport and metabolism to biofilm formation and infection severity are independent of *P. mirabilis* urease activity. (A) Representative phenol red urease activity curve over a 120-minute period of wild-type *P. mirabilis, argF::Lan*, or a urease mutant (*ureF::Lan*, negative control). (B-C) Urease activity curves of B) wild-type *P. mirabilis* and C) *argF::Kan* when incubated in cell-free culture supernatants from either wild-type *P. mirabilis*, wild-type *E. faecalis,* or *arcD::Tn*. Data represent the mean ± SD for at least three independent experiments with at least two replicates each.

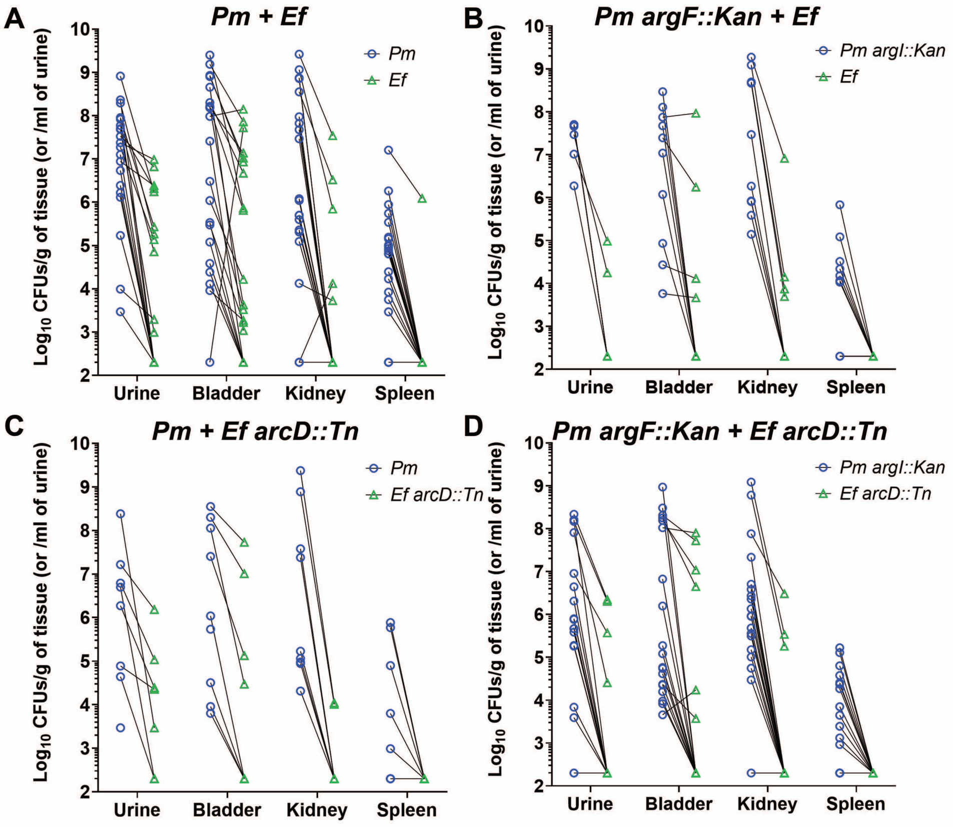

Supplemental 6. CFUs of *P. mirabilis* and *E. faecalis* from all coinfected mice. The CFUs of each species recovered from an individual coinfected mouse are connected with a black line for each organ. The inoculum for a particular group is labeled above the graph of interest.
