# Supplementary material for: Metabolic interplay between Proteus mirabilis and Enterococcus faecalis facilitates polymicrobial biofilm formation and invasive disease
Source: mBio. 2024 Oct 30;15(12):e02164-24. doi: 10.1128/mbio.02164-24 (PMC11640290; doi:10.1128/mbio.02164-24)
Supplement: Supplemental text — Supplemental methods. [file mbio.02164-24-s0002.pdf]

Supplemental Methods.

Growth curves. Overnight cultures of bacteria were adjusted to approximately 10^7 CFU/mL in indicated media. 200 µL of the adjusted bacterial suspension was distributed in at least triplicate wells of a clear 96-well plate and incubated in a BioTek Synergy H1 96-well plate reader at 37°C with continuous double-orbital shaking and a 1°C temperature differential between the top and bottom of the plate to prevent condensation. Bacterial growth was assessed via absorbance (OD_{600}) every 15 minutes for a period of 18 hours. For assessment of CFUs, 5 mL bacterial suspensions were incubated at 37°C with shaking at 225 RPM, aliquots were taken hourly, serially diluted, and plated onto appropriate agar for determination of CFUs. *Pm* minimal salts medium (PMSM) was used in experiments requiring defined growth medium (10.5 g/L K_2HPO_4, 4.5 g/L KH_2PO_4, 1 g/L $(NH_4)_2SO_4$, 15 g/L agar, supplemented with 0.002% nicotinic acid, 1 mM $MgSO_4$, and 0.2% glycerol). PMSM was supplemented further with addition of 10 mM L-ornithine, L-arginine, or L-citrulline as indicated. For co-challenge experiments, samples were plated on plain LSLB agar (total CFUs), LSLB with kanamycin (*Pm* mutant strain CFUs), and BHI agar supplemented with 100 µg/ml spectinomycin (*Ef* CFUs).

Biofilm fractionation for composition analysis. Biofilm suspensions were fixed for 1 hour with formaldehyde (37%) by incubating at room temperature with shaking at 200 RPM. 1 M NaOH was then added and samples were incubated for 3 hours at room temperature with shaking at 200 RPM. Samples were then centrifuged (20,000xg) for 1 hour at 4°C. Supernatant containing the soluble extrapolymeric substance (EPS) was removed and placed in a sterile microcentrifuge tube, while the remaining pellet was resuspended in 1 mL Milli-Q water to generate the cell fraction

(CF). The EPS was filtered through a 0.22 µm filter and then was transferred to Slide-A-Lyzer dialysis cassette (Thermofisher, Cat# 66380) and placed in beaker containing Milli-Q water. The Milli-Q water was replaced twice after 2 hours, after which the sample was left to dialyze overnight. Samples were removed from the dialysis cassette to generate the EPS fraction (EPS). All samples were stored at -20C until end point analysis. Total protein was determined via Pierce BCA protein assay kit (Thermofisher, Cat# 23250) following manufacturer instructions.

Proteomics methods. Single-species and polymicrobial biofilms were established in TSB-G in 24 well plates, washed gently with PBS, and suspended in PBS. A surfactant-aided precipitation/on-pellet digestion method was adopted in the current study for sample preparation [96]. In brief, 100 μg protein was aliquoted from each sample and diluted to 1 μg/μL with 1% SDS. Protein was sequentially reduced by 10 mM dithiothreitol (DTT) at 56°C for 30 min and alkylated by 25 mM iodoacetamide (IAM) at 37°C in darkness for 30 min. Both steps were performed with rigorous vortexing in a thermomixer (Eppendorf). A total of 6 volumes of chilled acetone was then added to each sample with constant vortexing, and the mixture was incubated at -20°C for 3 hr. After centrifugation at 20,000 g, 4°C for 30 min, liquid was decanted, and protein pellet was gently washed by 500 μL methanol and air-dried for 1 min. A volume of 80 μL 50 mM pH 8.4 Tris-formic acid (FA) was then added, and samples were sonicated to loosen the protein pellet. A total volume of 20 μL trypsin (Sigma Aldrich, dissolved in 50 mM pH 8.4 Tris-FA) was added for 6-hr digestion at 37°C with rigorous vortexing in a thermomixer. Digestion was terminated by addition of 1 μL FA, and samples were centrifuged at 20,000 g, 4°C for 30 min. Supernatant was carefully transferred to LC vials for analysis.

The LC-MS system consists of a Dionex μLtimate 3000 nano LC system, a Dinex μLtimate 3000 micro LC system with a WPS-3000 autosampler, and a ThermoFisher Orbitrap Fusion Lumos mass spectrometer. A large-inner diameter (i.d.) trapping column (300-um i.d. x 5 mm) was coupled to the nano LC column (75-um i.d. x 65 cm, packed with 2.5-um Xselect CSH C18 material) for high-capacity sample loading, cleanup and delivery. For each sample, 4 μL derived peptide was injected for LC-MS analysis. Mobile phase A and B were 0.1% FA in 2% acetonitrile (ACN) and 0.1% FA in 88% ACN. The 180-min LC gradient profile was: 4% for 3 min, 4–11 for 5 min, 11–32% B for 117 min, 32–50% B for 10 min, 50–97% B for 5 min, 97% B for 7 min, and then equilibrated to 4% for 27 min. The mass spectrometer was operated under data-dependent acquisition (DDA) mode with a maximal duty cycle of 3 s. MS1 spectra was acquired by Orbitrap (OT) under 120k resolution for ions within the m/z range of 400-1,500. Automatic Gain Control (AGC) and maximal injection time was set at 120% and 50 ms, and dynamic exclusion was set at 45 s, ± 10 ppm. Precursor ions were isolated by quadrupole using a m/z window of 1.2 Th, and were fragmented by high-energy collision dissociation (HCD). MS2 spectra was acquired OT under 15k resolution with a maximal injection time of 50 ms. Detailed LC-MS settings and relevant information are enclosed in a previous publication by Shen et al (1).

LC-MS files were searched against a NCBI protein sequence database containing both *Proteus mirabilis* and *Enterococcus faecalis* protein sequences using Sequest HT embedded in Proteome Discoverer 1.4 (ThermoFisher Scientific). Target-decoy searching approach using a concatenated forward and reverse protein sequence database was employed for global FDR estimation and control. Searching parameters include: 1) Precursor ion mass tolerance: 20 ppm; 2) Product ion mass tolerance: 0.02 Da; 3) Maximal missed cleavages per peptide: 2; 4) Fixed modifications: carbamidomethylation of cysteine; 5) Dynamic modifications: Oxidation of

methionine, Acetylation of peptide N-terminals. Peptide filtering, protein inference and grouping, and FDR control were accomplished by Scaffold v5.0.0 (Proteome Software, Inc.) The filtered peptide-spectrum match (PSM) list was exported. Protein quantification was performed using IonStar, an in-house developed MS1 ion current-based quantitative proteomics method (2). Peptide quantitative features were first generated by a two-step procedure encompassing 1) Chromatographic alignment with ChromAlign for inter-run calibration of retention time (RT) shift; ii) Data-independent MS1 feature generation a direct ion-current extraction (DICE) method, which extracts ion chromatograms for all precursor ions with corresponding MS2 scans in the aligned dataset with a defined m/z-RT window (10 ppm, 1 min). Both steps were accomplished in SIEVE v2.2 (ThermoFisher Scientific). Post-feature generation data processing was accomplished by UHR-IonStar v1.4 (https://github.com/JunQu-Lab/UHRIonStarApp) (3). The filtered PSM list and the quantitative features database were first integrated by MS2 scan number to generate a list of annotated frames with peptide sequence assignment. The annotated frames were then subjected to dataset-wide normalization, principal component-based detection and removal of peptide outliers, and data aggregation to protein level. Protein quantification results were exported and manually curated and processed in Microsoft Excel.

Glass Bladder CAUTI Model. Experiments in this model were conducted using artificial urine media (AUM) with 250 mM urea, following the published recipe (4). The glass bladder model consists of a 500 mL water-jacketed glass vessel (Chemglass Life Sciences CG-1929-06, Vineland, NJ) maintained at 37°C via a circulating water bath (Isotemp Refrigerated Circulator Model 901, Fisher Scientific) and capped with a #14 plug (Caplugs HUP14-04, Buffalo, NY). A size 14 French silicone Foley catheter (Dynarex, Blauvelt, NY) is aseptically inserted into the

vessel through an opening in the bottom, the balloon is inflated, and connected to a drainage bag (AMSure urinary drainage bag, Amsino, Pomona, CA), mimicking the setup that would be used for a patient (5, 6). AUM is supplied to the glass bladder at a constant flow rate (0.5-1.0mL/min) through a peristaltic pump (VWR MASTERFLEX pump, Radnor, PA) fed from a 2 liter Corning gl45 threaded bottle sealed with a #13.7 cap (Caplugs HUP13.7-04, Buffalo, NY) (4). A 10 mL inoculum of 10^8 CFUs of *Pm*, *Ef*, or a 1:1 mixture of the two uropathogens was introduced into the glass bladder and allowed to establish without flow for 1 hour before initiating flow. Samples were collected from the catheter port at 0, 3, 6, 9, 12, and 24 hours post inoculation to track bacterial CFUs. After 24 hours, the catheter was aseptically removed and sectioned as follows: the eyelet section was defined as 20 mm from the tip of the catheter to below the balloon, while the rest of the catheter was sectioned into 10 mm segments. The eyelet section and segments for crystal violet staining were dried for 15 minutes, placed into 1 mL of 0.1% crystal violet solution, stained for 10 minutes, gently washed twice with 1x PBS, and the lumen of the catheter segment was touched to a Kim wipe (Kimberly-Clark Corporation, Loudon, TN) to remove excess fluid. Stained sections were then placed in 1 mL of 95% ethanol in a 1.7ml tube, vortexed for 10 minutes, and crystal violet absorbance was read at 570 nm. To determine CFUs, catheter segments were wicked with a Kim wipe to remove excess fluid, washed twice in 1x PBS, placed in 1 mL of 1x PBS in a 1.7mL tube, vortexed for 10 minutes, serially diluted, and plated.

Urease activity assay. Urease activity in live, whole-cell *P. mirabilis* was assessed using a previously published alkalimetric assay (7). Briefly, *P. mirabilis* and Δ*argF* were cultured overnight in LSLB for ~16 hours 37°C with aeration, centrifuged to pellet, resuspended in sterile saline, and 20 μl of each suspension was added to wells of a 96-well plate containing 100 μl of

potassium phosphate buffer supplemented with 0.001% WT/vol phenol red and 500 mM urea. Optical density at 562 nm was measured every 60 seconds for 2 hours using a Synergy H1 (BioTek). For assessing enhancement of urease activity by *E. faecalis* cell-free supernatants, bacteria were cultured to mid-log phase (OD_{600}=0.5), centrifuged to pellet, and resuspended in potassium phosphate buffer. Resuspended cultures were incubated at 37°C with for 90 minutes, centrifuged to pellet, and supernatants were passaged through a 0.22µm pore size filter.

Murine model of CAUITI. CAUTI studies were performed as previously described (8–10). In short, the inoculum was prepared by washing overnight cultures in PBS, adjusting to ~2x10^8 CFU/mL, then diluting 1:100 to make a final inoculum of 2x10^6 CFU/mL. Co-challenge inocula were generated by combining a 50:50 mix of each single-species inoculum. Female CBA/J mice aged 5-8 weeks (Jackson Laboratory) were anesthetized with a weight appropriate dose of ketamine/xylazine (80-120mg/kg ketamine and 5-10 mg/kg xylazine) via IP injection, after which mice were inoculated transurethrally with 50 µL of the appropriate inoculum suspension, delivering ~1x10^5 CFU/mouse. A 4 mm segment of sterile silicone tubing (0.64 mm O.D., 0.30 mm I.D., Braintree Scientific Inc.) was advanced into the bladder during inoculation to be retained for the duration of the study as done previously (8, 11). After 96 hours, urine was collected, bladders, kidneys, and spleens were harvested and placed into 5 mL Eppendorf tubes containing 1 mL 1x PBS and 500 µL of 3.2mm stainless steel beads (Next Advance, Troy, NY). Tissues were homogenized using a Bullet Blender 5 Gold (Next Advance) at Speed 8 for 4 minutes. Bladders were treated to two cycles to ensure full homogenization. Tissue homogenates were serially diluted and plated onto appropriate agar using an EddyJet 2 spiral plater (Neutec Group) for determination of CFUs using a ProtoCOL 3 automated colony counter (Synbiosis).

Supplemental Methods References:

1. Ni J, Shen TCD, Chen EZ, Bittinger K, Bailey A, Roggiani M, Sirota-Madi A, Friedman ES, Chau L, Lin A, Nissim I, Scott J, Lauder A, Hoffmann C, Rivas G, Albenberg L, Baldassano RN, Braun J, Xavier RJ, Clish CB, Yudkoff M, Li H, Goulian M, Bushman FD, Lewis JD, Wu GD. 2017. A role for bacterial urease in gut dysbiosis and Crohn's disease. Science translational medicine 9.
2. Shen X, Shen S, Li J, Hu Q, Nie L, Tu C, Wang X, Orsburn B, Wang J, Qu J. 2017. An IonStar Experimental Strategy for MS1 Ion Current-Based Quantification Using Ultrahigh-Field Orbitrap: Reproducible, In-Depth, and Accurate Protein Measurement in Large Cohorts. 7. J Proteome Res 16:2445–2456.
3. Shen X, Shen S, Li J, Hu Q, Nie L, Tu C, Wang X, Poulsen DJ, Orsburn BC, Wang J, Qu J. 2018. IonStar enables high-precision, low-missing-data proteomics quantification in large biological cohorts. 21. Proc Natl Acad Sci U S A 115:E4767–E4776.
4. Brooks T, Keevil C w. 1997. A simple artificial urine for the growth of urinary pathogens. Letters in Applied Microbiology 24:203–206.
5. Stickler DJ, Morris NS, Winters C. 1999. Simple physical model to study formation and physiology of biofilms on urethral catheters. Methods Enzymol 310:494–501.
6. Nzakizwanayo J, Pelling H, Milo S, Jones BV. 2019. An In Vitro Bladder Model for Studying Catheter-Associated Urinary Tract Infection and Associated Analysis of Biofilms. Methods Mol Biol 2021:139–158.

7. Keogh D, Tay WH, Ho YY, Dale JL, Chen S, Umashankar S, Williams RBH, Chen SL, Dunny GM, Kline KA. 2016. Enterococcal Metabolite Cues Facilitate Interspecies Niche Modulation and Polymicrobial Infection. 4. Cell Host Microbe 20:493–503.
8. Armbruster CE, Smith SN, Johnson AO, DeOrnellas V, Eaton KA, Yep A, Mody L, Wu W, Mobley HLT. 2017. The Pathogenic Potential of Proteus mirabilis Is Enhanced by Other Uropathogens during Polymicrobial Urinary Tract Infection. 2. Infect Immun 85.
9. Brauer AL, Learman BS, Taddei SM, Deka N, Hunt BC, Armbruster CE. 2022. Preferential catabolism of l- vs d-serine by Proteus mirabilis contributes to pathogenesis and catheter-associated urinary tract infection. Mol Microbiol 118:125–144.
10. Smith SN, Armbruster CE. 2019. Indwelling Urinary Catheter Model of Proteus mirabilis Infection. Methods Mol Biol 2021:187–200.
11. Guiton PS, Hung CS, Hancock LE, Caparon MG, Hultgren SJ. 2010. Enterococcal Biofilm Formation and Virulence in an Optimized Murine Model of Foreign Body-Associated Urinary Tract Infections. Infect Immun 78:4166–4175.
